# Inhibition of invasive salmonella by orally administered IgA and IgG monoclonal antibodies

**DOI:** 10.1371/journal.pntd.0007803

**Published:** 2020-03-23

**Authors:** Angelene F. Richards, Jennifer E. Doering, Shannon A. Lozito, John J. Varrone, Graham G. Willsey, Michael Pauly, Kevin Whaley, Larry Zeitlin, Nicholas J. Mantis

**Affiliations:** 1 Department of Biomedical Sciences, University at Albany School of Public Health, Albany, New York, United States of America; 2 Division of Infectious Diseases, Wadsworth Center, New York State Department of Health, Albany, New York, United States of America; 3 Mapp Biopharmaceutical, San Diego, California, United States of America; University of Colorado Health Sciences Center, UNITED STATES

## Abstract

Non-typhoidal *Salmonella enterica* strains, including serovar Typhimurium (STm), are an emerging cause of invasive disease among children and the immunocompromised, especially in regions of sub-Saharan Africa. STm invades the intestinal mucosa through Peyer’s patch tissues before disseminating systemically. While vaccine development efforts are ongoing, the emergence of multidrug resistant strains of STm affirms the need to seek alternative strategies to protect high-risk individuals from infection. In this report, we investigated the potential of an orally administered O5 serotype-specific IgA monoclonal antibody (mAb), called Sal4, to prevent infection of invasive *Salmonella enterica* serovar Typhimurium (STm) in mice. Sal4 IgA was delivered to mice prior to or concurrently with STm challenge. Infectivity was measured as bacterial burden in Peyer’s patch tissues one day after challenge. Using this model, we defined the minimal amount of Sal4 IgA required to significantly reduce STm uptake into Peyer’s patches. The relative efficacy of Sal4 in dimeric and secretory IgA (SIgA) forms was compared. To assess the role of isotype in oral passive immunization, we engineered a recombinant IgG1 mAb carrying the Sal4 variable regions and evaluated its ability to block invasion of STm into epithelial cells *in vitro* and Peyer’s patch tissues. Our results demonstrate the potential of orally administered monoclonal IgA and SIgA, but not IgG, to passively immunize against invasive Salmonella. Nonetheless, the prophylactic window of IgA/SIgA in the mouse was on the order of minutes, underscoring the need to develop formulations to protect mAbs in the gastric environment and to permit sustained release in the small intestine.

## Author summary

The bacterium *Salmonella enterica* is responsible for significant morbidity and mortality in the developed and developing worlds. While the pathogen is most renowned as the causative agent of typhoid fever, the emergence of invasive non-typhoid strains like *S*. *enterica* serovar Typhimurium (STm) are of great concern because of their propensity to cause severe disease in children under the age of five. In this report, we demonstrate in a mouse model that oral administration of a monoclonal antibody targeting the surface of STm is able to prevent the bacterium from infecting gastrointestinal tissues, the first step in the dissemination process. We show that IgA antibodies (which are normally found in the gut) were far superior than an equivalent IgG antibody (normally found in blood) at defending the intestine from STm infection. These results lay the foundation for future studies aimed at the development of an orally administered antibody cocktail capable of providing temporary immunity to pathogens like *S*. *enterica*.

## Introduction

Enteric bacterial pathogens constitute a major burden on global health, especially in children younger than five years of age [1, 2]. The Global Enteric Multicenter Study (GEMS) surveyed children ages 0–59 months in seven countries in sub-Saharan Africa and south Asia and identified the leading etiological agents of moderate-to-severe (MSD) and less-severe diarrhea (LSD) in this age group [1, 3]. Included on the list were *Shigella* species, *Campylobacter jejuni*, *Vibrio cholerae* and enterotoxigenic *E*. *coli* (ETEC) among others. Episodes of MSD and LSD can each have long term impacts on child health, most notably linear growth faltering. Other pathogens like multidrug resistant typhoid and invasive non-typhoidal Salmonella (iNTS) caused by different serovars of *Salmonella enterica* are also a source of infections in sub-Saharan Africa that can have short- and long-term health consequences [4–6]. The dire need for vaccines against enteric bacterial pathogens like *C*. *jejuni*, *Shigella*, ETEC and *Salmonella* has been recognized for decades, especially within military settings [2]. While there are numerous candidate vaccines under evaluation, the path forward remains challenging and alternative approaches need to be considered to combat *C*. *jejuni*, *Shigella*, ETEC and *Salmonella* in the immediate future.

With the advent of affordable and scalable platforms for the production of pathogen-specific IgG and secretory IgA (SIgA), the notion of oral passive immunization with polyclonal or monoclonal antibodies (mAbs) as a strategy to blunt diarrheal diseases in high-risk populations is gaining attention. For example, it was reported that ingestion of polyclonal hyperimmune bovine colostrum (HBC), marketed as Travelan, reduces experimental traveler’s associated ETEC infection [7]. Sears and colleagues recently presented evidence that IgG and possibly IgA antibodies in Travelan and a related HBC product (IMM-24E) may exert their protective effects through arresting ETEC motility and complement-mediated killing [8]. More recently, Guintini and colleagues demonstrated that oral administration of IgG or IgA mAbs targeting a single adhesin (CfaE) were able to reduce ETEC colonization by several orders of magnitude in a mouse model [9]. In the case of invasive *Salmonella enterica* serovar Typhimurium (STm), Corthésy and colleagues reported that polyreactive secretory-like IgA/IgM mixtures were capable of reducing bacterial entry into Peyer’s patch tissues [10, 11]. While these studies represent a proof of concept that oral immunoglobulins can abrogate Salmonella infection, the amount of IgA/IgM required to achieve a reduction in bacterial burden was excessive (*i*.*e*., ~10 mg of SIgA/IgM; ~ 500 mg/kg) and likely impractical if translated to a human setting. For that reason, we sought to investigate the potential benefit of a mAb-based passive immunization approach in blocking invasive *Salmonella*.

Sal4 is a well-characterized, dimeric IgA mAb originally isolated from a panel of B cell hybridomas derived from Peyer’s patch tissues of mice that had been immunized with an attenuated strain of STm [12]. Sal4 recognizes the O5-antigen of STm lipopolysaccharide (LPS) [13]. The O-antigen of STm is a tetrasaccharide consisting of galactose, rhamnose, and mannose, with an abequose (3,6 dideoy-galactose) moiety on the mannose side chain. The O5 antigen is conferred when the abequose residue is acetylated, while the O4 antigen is defined by the absence of acetylation modification [14]. Both STm O4 and O5 serotypes are invasive in mouse models of intragastric and parenteral challenge, although the actual lethal dose values vary slightly [13].

In the so-called backpack tumor model, it was shown that Sal4 IgA, when actively transported into the intestinal lumen of mice in the form of secretory IgA (SIgA), was able to reduce STm uptake into Peyer’s patch tissues [13]. Peyer’s patches represent the point of entry for invasive strains of *Salmonella enterica* and the bottleneck for systemic dissemination [15]. Sal4 IgA’s protective capacity was limited to the gut, as even high levels of Sal4 IgA in circulation were unable to curtail STm systemic infection in the face of a parenteral bacterial challenge [13]. Thus, Sal4 IgA limits STm infection exclusively in the context of the gastrointestinal lumen. Although the exact mechanisms by which Sal4 IgA prevents bacterial uptake into Peyer’s patch tissues have not been fully resolved, Sal4 IgA strongly promotes bacterial agglutination *in vitro* and is a potent inhibitor of STm flagella-based motility in liquid and viscous media [16].

In this report, Sal4 IgA was chosen as a prototype to investigate the potential of orally administered mAbs to passively immunize against invasive Salmonella. We first established a robust mouse model of bacterial entry into Peyer patch tissues and then used the model to compare the efficacy of Sal4 as dimeric IgA, secretory IgA and even IgG, in limiting bacterial access to the intestinal mucosa. We also generated and characterized a second O5-specific IgA mAb and compared it to Sal4 IgA *in vitro* and *in vivo*.

## Methods

### Ethics statement

The mouse experiments described in this study were reviewed and approved by the Wadsworth Center’s Institutional Animal Care and Use Committee (IACUC) under protocol #17–428. The Wadsworth Center complies with the Public Health Service Policy on Humane Care and Use of Laboratory Animals and was issued assurance number A3183-01. The Wadsworth Center is fully accredited by the Association for Assessment and Accreditation of Laboratory Animal Care (AAALAC). Obtaining this voluntary accreditation status reflects that Wadsworth Center’s Animal Care and Use Program meets all standards required by law and goes beyond the standards as it strives to achieve excellence in animal care and use. Mice were euthanized by carbon dioxide asphyxiation followed by cervical dislocation, as recommended by the Office of Laboratory Animal Welfare (OLAW), National Institutes of Health.

### Bacterial strains and growth conditions

*Salmonella enterica* serovar Typhimurium (STm) strains used in this study are described in **Table 1**. STm ATCC 14028 was purchased from the American Type Culture Collection (Manassas, VA) [17]. *S*. Typhimurium strains AR04 (*zjg8101*::*kan oafA126*::Tn*10d-*Tc *fkpA-lacZ)* and AR05 (*zjg8101*::*kan*) are derivatives of ATCC 14028, as described [16, 18]. Unless otherwise stated, single colonies were used to inoculate sterile Luria-Bertani (LB) broth and incubated overnight at 37°C with aeration, then subcultured in fresh LB to mid-log phase (OD_600_ 0.40) before use.

**Table 1:** ST strains and derivatives used in this study.

Strain	O-Ag	genotype	X-Gal	Reference
ATCC14028	O5	wild type	W	[17]
AR05	O5	*zjg8101*::*kan*	W	[18]
AR04	O4	*zjg8101*::*kan oafA126*::Tn*10d-*Tc *fkpA-lacZ*	B	[18]
CS022	O5	*pho-24* (PhoP constitutive)	W	[19]
SJF59	O4	*phoP-24 oafA126*:: Tn*10d-Tc*	W	This study

### Monoclonal antibodies (mAbs) and hybridomas

Antibodies used in this study are listed in **Table 2**. The B cell hybridoma cell lines secreting the monoclonal polymeric IgA antibody, Sal4, specific for O5-antigen and 2D6 IgA, specific for *V*. *cholerae* Ogawa LPS, were originally obtained from Dr. Marian Neutra (Children’s Hospital Boston) [13]. Purified dimeric Sal4 IgA (dIgA) and recombinant human secretory component (rSC) were associated for 1 h at room temperature to generate Sal4 SIgA, as described [20]. Chimeric Sal4 IgG1 and PB10 IgG1, specific for ricin toxin, were provided by Mapp Biopharmaceutical (San Diego, CA). 2D6 IgA and PB10 IgG1 mAbs were used as IgA and IgG1 isotype controls respectively throughout the study. The PeA3 murine B cell hybridoma secreting a monoclonal IgA against the STm O5 antigen was generated from the Peyer’s patches of BALB/c mice repeatedly immunized orally with STm, essentially as described [13]. The resulting hybridomas were screened by ELISA for reactivity with STm whole cells and purified LPS (Sigma-Aldrich, St. Louis, MO).

**Table 2:** Anti-ST and control mAbs used in this study.

Antibody	species	form	source	epitope
Sal4	mouse	IgA	hybridoma	STm O5 Ag
Sal4	mouse	SIgA	hybridoma	STm O5 Ag
Sal4	chimeric	IgG1	Nicotiana	STm O5 Ag
PeA3	mouse	IgA	hybridoma	STm O5 Ag
2D6	mouse	IgA	hybridoma	*V*.*cholerae* LPS
2D6	chimeric	IgG	Nicotiana	*V*.*cholerae* LPS

### Mice

Female BALB/c mice aged 8–12 weeks were obtained from Taconic Biosciences (Rensselaer, NY) and cared for by the Wadsworth Center Animal Core Facility. All experiments were performed in strict accordance with protocols approved by the Wadsworth Center’s IACUC, as noted above.

### Enzyme-linked immunosorbent assays (ELISAs)

For direct ELISAs, 96-well NUNC MaxiSorp plates (ThermoScientific, Waltham, MA) were coated with 0.1 ml of STm LPS (1 μg/ml in sterile PBS) overnight at 4°C. Wells were blocked with PBS containing 0.1% Tween-20 (PBST) and 2% goat serum for 2 h at room temperature before washing with PBST. Plates were developed using goat anti-mouse and goat anti-human HRP-conjugated secondary IgG antibodies (final concentration of 0.5 μg/ml) and SureBlue TMB Microwell Peroxidase Substrate (KPL, Gaithersburg, MD). For the whole-bacteria ELISA, 96-well NUNC MaxiSorp plates were coated with poly-L-lysine (10 μg/ml) overnight at 4°C. Overnight cultures of STm were washed twice with PBS and then placed into each well of the microtiter plate. The plates were centrifuged two times at 500 x *g* for 3 min (rotating 180° for the second spin), and then fixed with 2% paraformaldehyde (PFA) in PBS. The bacteria-coated plate was then treated with sterile glycine (0.1 M) to quench residual PFA and ELISAs were performed as described above. All plates were read by spectrophotometry (A_450_) within 30 minutes of developing using a VersaMax microplate reader and SoftMaxPro 5.2 software.

### Soft agar motility assays

For the soft agar motility assays, LB medium with 0.3% Bacto agar (Becton Dickinson) was prepared with 15 μg/ml of each mAb of interest poured into 60 mm Petri dishes (*n* = 3 per treatment group) and allowed to set at room temperature for 30 min. Individual colonies of ATCC 14028 STm were then picked from a freshly-streaked LB agar plate and stabbed directly into the center of the plate [21]. The plates were placed in a 37°C incubator and the diameters of the concentrically growing bacterial cultures were measured at 60-minute intervals.

### Bacterial agglutination assays

An aliquot of overnight liquid cultures of STm (100 μl) were mixed in equal ratio (v/v) with PBS containing a final concentration of 15 μg/ml of each IgA mAb of interest (*n* = 6 per treatment), and then placed into individual wells of a U-bottom 96 well plate. The plate was incubated at 37°C and visually monitored every 15 minutes for clumping of cells, as described [16].

### HeLa cell invasion assay

HeLa cells were obtained from the ATCC and maintained in Dulbecco’s Modified Eagle Media (DMEM) with 10% fetal bovine serum at 37°C and 5% CO_2_. Cells were seeded at 5 x 10^5^ cells/mL in 96-well plates and grown for 24 to 36 h to establish 70–90% confluency. Prior to STm infection assays, HeLa cells were washed three times with serum-free DMEM. Overnight cultures of AR05 and AR04 were subcultured in LB at 37°C with aeration and adjusted to an OD_600_ of ~0.7. Strains were mixed 1:1 and washed twice by centrifugation (6,000 x g for 2 minutes) and resuspended in PBS. Bacteria were then diluted 1:10 in Hank’s Balanced Salt Solution (HBSS, Wadsworth Center Media Core) and an aliquot was plated on LB agar supplemented with kanamycin (50 μg/mL) and X-gal (40 μg/mL) to compute bacterial input. For the invasion assay, bacterial mixtures were incubated with 15 ug/mL of either 2D6 IgA, PeA3 IgA, or Sal4 IgA mAb for 15 min at 37°C to minimize agglutination. Treated bacteria were applied to HeLa cell monolayers and centrifuged at 1,000 x g for 10 min (rotating the plate 180° at 5 min) to promote STm adherence to HeLa cell surfaces. The microtiter plates were then incubated for 90 minutes at 37°C. Cells were washed three times with HBSS and treated with gentamicin (40 μg/mL) to eliminate extracellular bacteria. Finally, cells were washed with HBSS lysed with 1% Triton X-100 (in Ca^2+^ and Mg^2+^-free PBS), serially diluted, plated on LB agar containing kanamycin (50μg/mL) and X-gal (5-bromo-4-chloro-3-indolyl-β-D-galactopyranoside) (40μg/mL) and incubated overnight at 37°C. The competitive index [(%strain A recovered/%strain B recovered)/(%strain A inoculated/%strain B inoculated)] was calculated for each treatment group.

### STm intragastric challenge

Overnight cultures of AR05 and AR04 were subcultured to an OD_600_ of 0.7, combined 1:1 (v/v) and resuspended in PBS. An aliquot was plated on LB agar containing kanamycin (50 μg/mL) and X-gal (40 μg/mL) at the start of the experiment to determine bacterial input (CFUs). Before gavage, bacteria (~4 x 10^7^ CFUs) were either incubated for 10 minutes with mAbs (30 μg/mL, unless stated otherwise), or mAbs were provided as a “chase” immediately before STm gavage (50 μg/mouse, unless stated otherwise). Twenty-four hours later, the mice were euthanized by CO_2_ asphyxiation followed by cervical dislocation. For each mouse, laparotomy was performed, and the small intestine was removed above the cecum. Peyer’s patches from each mouse were pooled and placed in 1 mL sterile PBS on ice. Samples were then homogenized with a Bead Mill 4 Homogenizer (Fisher Scientific) three times for 30 seconds each. Homogenates were serially diluted, plated on LB agar containing kanamycin and X-Gal and incubated overnight at 37°C. Blue and white colonies were enumerated and the competitive indices (CI) were calculated as CI = [(% strain A recovered/% strain B recovered)/(% strain A inoculated/% strain B inoculated)]. Whole-plate dilutions (100 μl per plate) were required to observe enough colonies to calculate competitive indices. All samples that contained less than 30 CFUs (per 100 μl) were eliminated from the data set and considered “too few to count” (TFC). This pool of cells is more likely a representation of only a few bacteria that have successfully invaded and replicated within the lymphoid tissues, as Peyer’s patch entry by *Salmonella* has been shown the bottleneck for further dissemination during infection [15].

### Mouse model of STm systemic challenge

BALB/c female mice were administered 40 μg or 10 μg (unless otherwise indicated) of Sal4 IgA, Sal4 IgG1, or isotype control antibody in sterile PBS by intraperitoneal (i.p.) injection. An overnight culture of wildtype STm (ATCC 14028) was subcultured, washed in sterile PBS, and diluted to a final concentration of 5 x 10^4^ CFUs/mL. Twenty-four hours after passive immunization, mice were challenged with STm inoculum (1 x 10^4^ CFUs) by i.p. injection. An additional 24 h later, mice were euthanized by CO_2_ inhalation followed by cervical dislocation and spleens and livers were collected, weighed, and homogenized in 1 mL sterile PBS, as described above. Homogenates were serially diluted, plated on LB agar and incubated overnight at 37°C. Total CFUs were counted and computed for Log_10_ CFUs/gram (tissue).

### *In vitro* digestion assay

Sal4 IgA or Sal4 IgG mAbs were diluted to a final concentration of 0.1 mg/mL in simplified adult simulated gastric fluid (94 mM NaCl, 13 mM KCl; pH adjusted to 3.0 with 1M HCl) with or without pepsin (2000U/mL) on ice similarly as described [22–24]. Samples were incubated statically at 37°C and aliquots were taken after 10 minutes, 30 minutes, and 60 minutes of incubation and neutralized on ice to a pH of 7.0 to 7.4 using 1M NaOH. Following neutralization, all samples were analyzed for binding of purified STm LPS by ELISA as previously described. Concentrations of Sal4 IgA and Sal4 IgG were quantified by establishing a standard curve using SoftMax Pro 5.2.

## Results

### Oral administration of Sal4 IgA prevents infection of STm in mice

To explore the benefit of passively administered Sal4 IgA on reducing the invasion of Peyer’s patch tissues by STm, we developed a competitive infection assay using two STm strains, AR05 and AR04 [18]. AR05 is a kanamycin resistant derivative of the type strain ATCC 14028 (**Table 1**) that expresses the O5 antigen (O5-Ag). AR04 is a derivative of AR05 with a Tn*10* insertion in the acetyl transferase gene (*oafA126*::Tn*10d-*Tc) that abolishes the bacterium’s ability to express the O5 Ag. Therefore, Sal4 IgA reacts with AR05 but not AR04 (**S1 Fig**) [14, 16]. In addition, AR04 constitutively expresses β-galactosidase. Thus, AR05 (“white”) is readily distinguished from AR04 (“blue”) on when colonies grown on LB/X-Gal agar (**S2 Fig**). The competitive index (CI) is simply the ratio of AR05 to AR04 in the inoculum compared to the ratio of AR05 to AR04 recovered from Peyer’s patch tissues, that allows for normalization in variations of challenge inoculum and Peyer’s patch number between mice (see Materials and Methods).

Adult BALB/c mice were challenged by gavage with a 1-to-1 mixture of AR05 and AR04 and 24 h later Peyer’s patches were collected along the entire length of the small intestine. It should be noted that at this time point, there was no gross evidence of inflammation or secretory diarrhea. Peyer’s patch tissues were normal in size and number and there was no evidence of fluid accumulation in the intestinal lumen. These observations are consistent with what has been reported in the literature [25–27].

**Fig 1:**
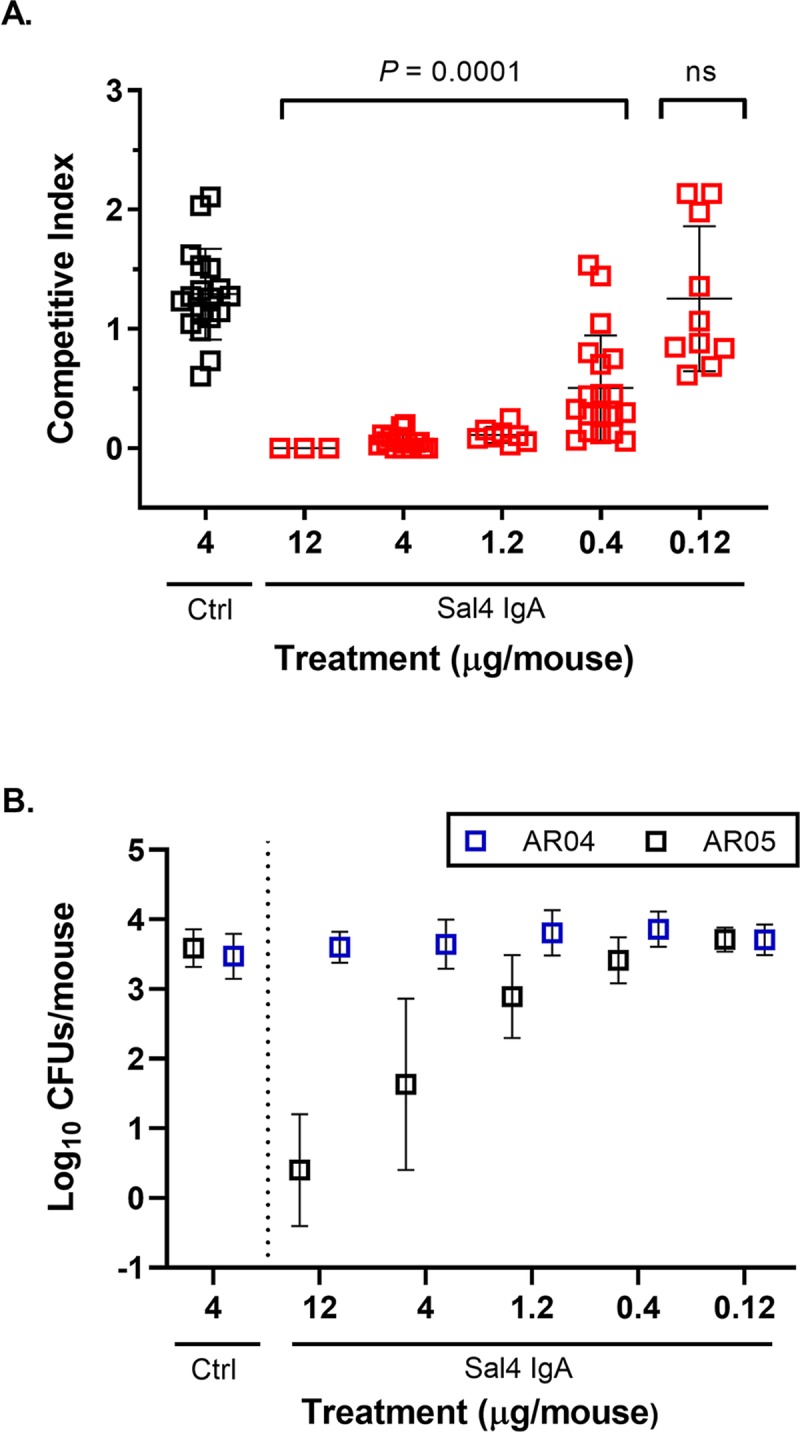
Orally administered Sal4 IgA blocks STm invasion into mouse Peyer’s patches. Adult BALB/c mice were challenged by gavage with a one-to-one mixture of STm O5 and O4 strains STm (4 x 10^7^ CFU total) co-administered with Sal4 IgA or an isotype control. Peyer’s patches were collected ~24 h later and assessed for bacterial loads. (A) Competitive indices and (B) total CFUs of AR05 and AR04 STm. Shown are the combined results of five independent experiments with at least 4 mice per group. Each symbol represents an individual mouse. Statistical significance evaluated for each concentration over the isotype control, as determined by Kruskal-Wallis test and Dunn’s post-hoc test.

The Peyer’s patch tissues were pooled and homogenized and the resulting homogenates were serially diluted onto LB agar containing kanamycin and X-Gal. Preliminary studies determined that an inoculum of 4 x 10^7^ total CFUs (1:1 AR05 and AR04), which is roughly equivalent to 20 x LD_50_ [13], resulted in the reproducible recovery of 10^2^−10^4^ CFUs from each mouse. The experiments also revealed AR05 was slightly more invasive than AR04, as evidenced by a CI of ~1.2 to 1.5. This minor difference in invasiveness between the two strains was only observable after computing the CI, as the total number of AR04 and AR05 CFUs in the Peyer’s patches were not significantly differ from each other (**Fig 1**). Furthermore, STm invasion of both AR05 and AR04 strains was confined to Peyer’s patch tissues. In a representative study, we found that there were 208 CFUs/mm of Peyer’s patch tissue, as compared to 0.1 CFUs/mm of proximal small intestine and 0.17 CFUs/mm distal small intestine. This ~2000 fold enrichment of STm in Peyer’s patch tissues is consistent bacterial uptake occurring primarily via M cells and agrees with what has been reported in the literature dating back almost 40 years [25, 28, 29].

We next examined the impact of Sal4 IgA on the ability of STm to invade Peyer’s patch tissues. Pre-treatment of the STm inoculum with Sal4 IgA resulted in a dose-dependent reduction in the number of AR05 recovered in Peyer’s patch tissues (**Fig 1**). The highest dose of Sal4 IgA tested (12 μg) resulted in >4 log_10_ reduction in AR05 invasion efficiency, as compared to controls. In contrast, the number of AR04 CFUs recovered from the same Peyer’s patch tissues was unaffected by Sal4 IgA treatment. The relative impact of Sal4 IgA on AR05 versus AR04 on Peyer’s patch invasion was most apparent when the recovery values were expressed as a CI (**Fig 1**). By this metric, the addition of as little as 0.4 μg of Sal4 IgA rendered AR05 at a competitive disadvantage (**Fig 1**). The addition of greater amounts of Sal4 further reduced the CI with a maximal reduction occurring at concentrations above 1.2 μg Sal4 IgA. Invasion of Peyer’s patch tissues by AR05 and AR04 was unaffected by 2D6, an anti-*Vibrio cholerae* IgA mAb that served as the isotype control for these studies [13, 21].

### Inhibition of STm invasion of epithelial cells *in vitro* and Peyer’s patch tissues *in vivo* by a second O5-specific IgA

A number of important biological activities have been ascribed to Sal4 IgA that likely contribute to its ability to limit bacterial uptake into Peyer’s patch tissues [12–14, 16, 18, 30]. Most notability is Sal4 IgA’s ability to arrest bacterial motility in liquid and semi-solid agar [16]. In addition, Sal4 IgA blocks *Salmonella* pathogenicity island 1 (SPI-1) type III secretion system (T3SS)-mediated entry of STm into epithelial cells and limits the translocation of T3SS effector proteins SlrP and SopB [18]. However, because Sal4 IgA is the only O5-specific IgA mAb that has been characterized in detail, it is not known these activites are unique to Sal4 shared by other O5-specific IgA antibodies. For that reason, we sought to generate additional O5-specific mouse IgA mAbs and evaluate them *in vitro* and *in vivo*. To this end, groups of BALB/c mice were immunized with an attenuated STm mutant [13]. B cell hybridomas were generated from Peyer’s patch lymphocytes from immunized mice and screened by ELISA for IgA reactivity with STm strain 14028. Despite numerous attempts, only a single stable B cell hybridoma secreting an O5-specific IgA mAb was identified, which we designated PeA3. PeA3 IgA bound STm LPS by ELISA (**S3 Fig**). In liquid culture, PeA3 promoted agglutination of AR05, but not AR04, thereby demonstrating its specificity for the O5 epitope (**S3 Fig**). Finally, PeA3 IgA significantly impeded STm flagella-based motility through soft agar (**S3 Fig**).

**Fig 2:**
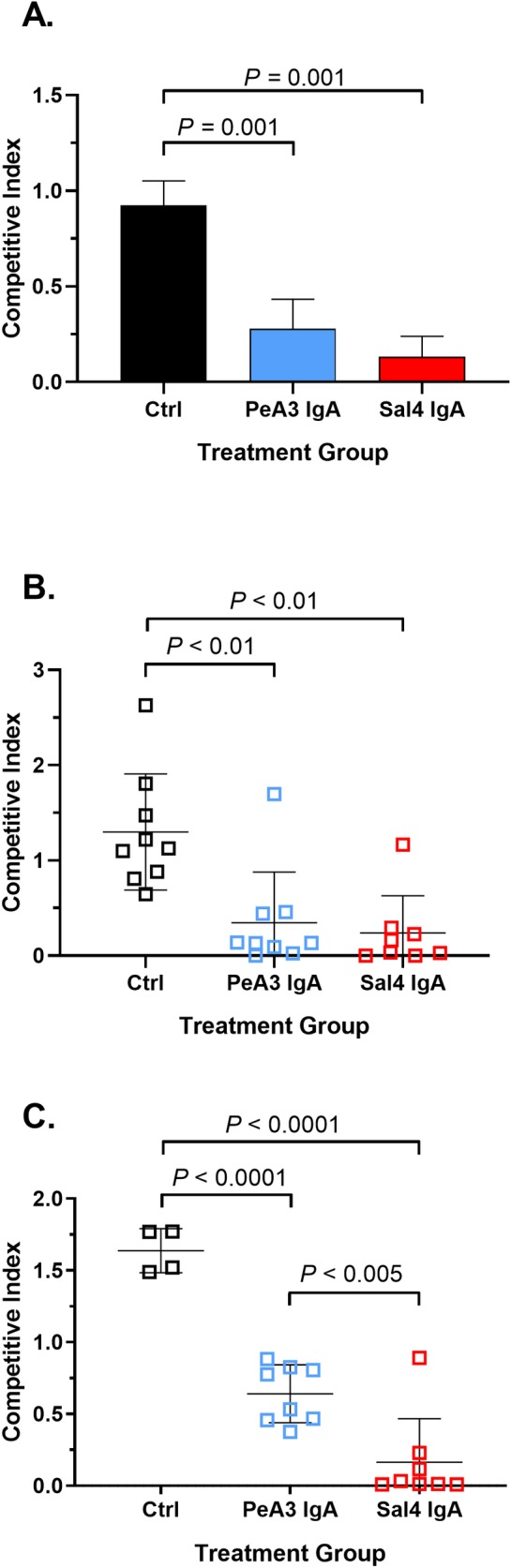
PeA3 IgA blocks wildtype STm invasion *in vitro* and *in vivo*. (A) A 1:1 mixture of AR04 and AR05 STm strains was treated with Sal4 IgA, PeA3 IgA, or isotype control antibody and applied to HeLa cells. Monolayers were then treated with gentamicin to eliminate extracellular bacteria and HeLa cells were lysed. The remaining cell lysate was enumerated for CFUs (*n* = 3 experiments, each done in triplicate). Panels B, C: Adult BALB/c mice were challenged by gavage with a 1:1 mixture of STm AR04 and AR05 O5 (4 x 10^7^ CFU total) in the present of 30 μg/mL (B) or 10 μg/mL (C) of indicated mAb. The mice were euthanized 24 h later and Peyer’s patches were assessed for bacterial loads. Shown are the combined results of two independent experiments with at least 4 mice per group. Statistical significance evaluated for each concentration over the isotype control, as determined by one-way ANOVA with either Dunnett’s (A, B) or Tukey’s (C) post-hoc tests.

To test whether PeA3 IgA impedes STm T3SS-mediated invasion into epithelial cells, a 1:1 mixture of AR05 and AR04 was treated with PeA3 IgA, Sal4 IgA, or an isotype control (2D6) before being applied to HeLa cells with gentle centrifugation to bypass the need for motility [16]. At the doses of mAb tested, PeA3 IgA treatment resulted in a significant reduction in AR05 uptake into HeLa cells (**Fig 2**).

To assess the ability of PeA3 IgA to block STm entry into mouse Peyer’s patch tissues, groups of BALB/c mice were challenged with a 1:1 mixture of AR05 and AR04 supplemented with a high (12 μg) or low (4 μg) dose of PeA3 IgA. As a control, Sal4 IgA was included in the experiments at the same doses as PeA3. At the high dose, PeA3 and Sal4 IgA were equally effective at blocking bacterial uptake into Peyer’s patch tissues, as evidenced by similar competitive indices (**Fig 2**). At the low dose, however, Sal4 IgA was ~five-fold more efficient than PeA3, as evidenced by mean CI values of 0.16 (± 0.30) versus 0.64 (± 0.20), respectively (**Fig 2**). The differences in mAb activities *in vivo* likely reflect different relative avidities of Sal4 and PeA3 for STm LPS. Nonetheless, these data demonstrate that another O5-specific IgA mAb, besides Sal4, is able to inhibit STm invasion of epithelial cells *in vitro* and Peyer’s patch tissues *in vivo*.

### Pre-exposure prophylactic activity of Sal4 IgA

Given that human breast milk provides passive immunity to newborns primarily in the form of specific polyclonal SIgA [31], there is interest in replicating this phenomenon utilizing mAbs for protection against enteric pathogens. *In vivo*, antigen-specific oral IgA has already been shown to protect against rotavirus challenge in a suckling mouse model [32] and oral feedings of recombinant VHH-IgAs, specific for the F4 fimbriae of ETEC, to piglets prior to bacterial challenge significantly reduced disease progression compared to control-fed groups [33]. After establishing Sal4 as a superior anti-O5 mAb, we sought to investigate its potential prophylactically in a model of STm infection.

**Fig 3:**
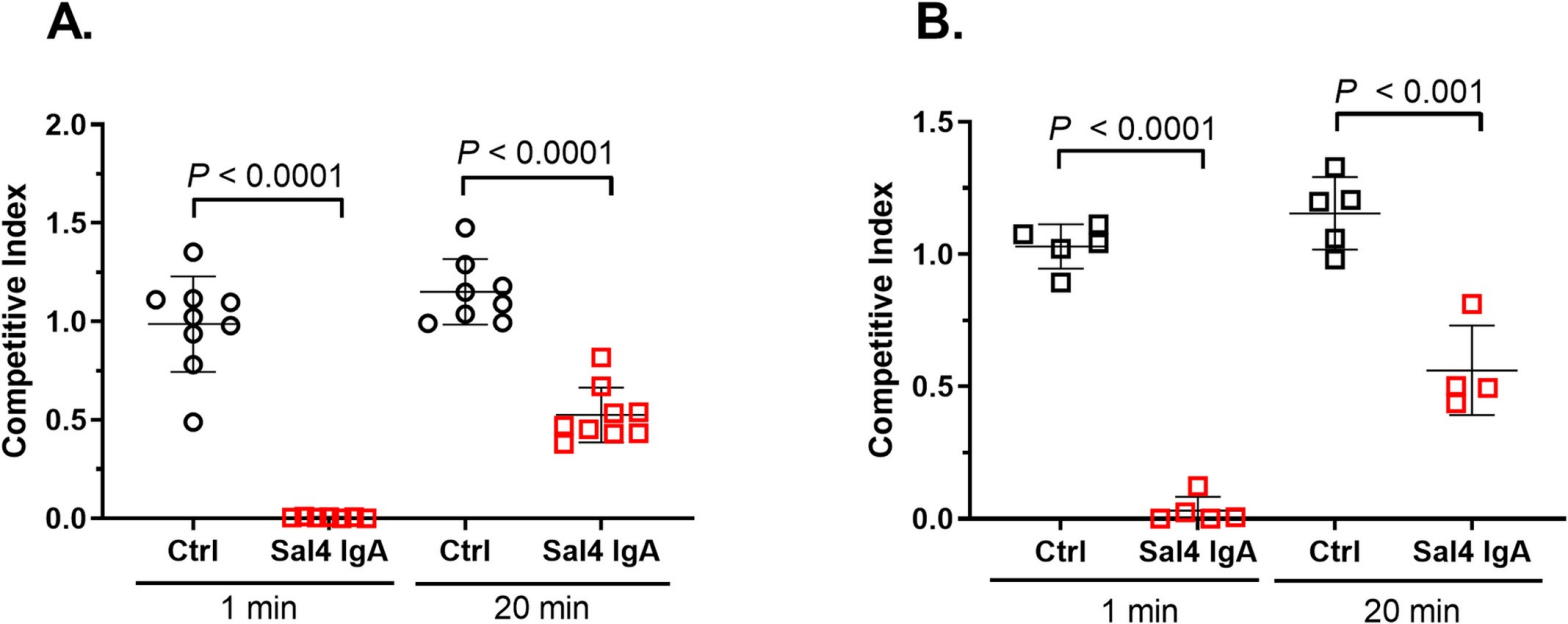
Sodium bicarbonate and protease inhibitors improve Sal4 IgA prophylactic activity. Adult BALB/c mice were gavaged with Sal4 IgA (50 μg) in (A) 3% NaHCO_3_ or (B) a protease inhibitor cocktail 20 min or 1 min before STm challenge. The mice were euthanized 24 h later and Peyer’s patches were assessed for bacterial loads, as a readout of bacterial invasion. Shown are the results of three independent experiments with at least 5 mice per group. Each symbol represents an individual mouse. Statistical significance compared to the isotype control at each time point, as determined by unpaired Student’s *t*-test.

We first addressed the degree to which Sal4 IgA administered to mice in advance of ST challenge retained its capacity to limit AR05 uptake into Peyer’s patch tissues. Sal4 IgA was delivered by gavage to mice 20 min prior to challenge with 1:1 mixture of AR05 and AR04. Under these conditions, Sal4 IgA’s activity was effectively lost. For example, Sal4 IgA (36 μg) given to mice by gavage 20 min before STm challenge afforded no protection against Peyer’s patch uptake (**S4 Fig**). In an effort to overcome the possible deleterious effects of gastric pH and intestinal proteases like pepsin, the experiments were repeated with the addition of sodium bicarbonate (3% NaHCO_3_) or protease inhibitors. The co-administration of Sal4 IgA with either sodium bicarbonate or protease inhibitors resulted in a 40–50% reduction in AR05 uptake into the Peyer’s patches (**Fig 3**), consistent with pH and proteases as being factors influencing the half life of Sal4 IgA in the gastric and intestinal lumen. While beyond the scope of the current study, these results underscore the necessity of identifying formulations capable of protecting orally administered antibodies like Sal4 IgA from the gastric environment.

### Benefit of SC on Sal4 IgA function in the mouse model

**Fig 4:**
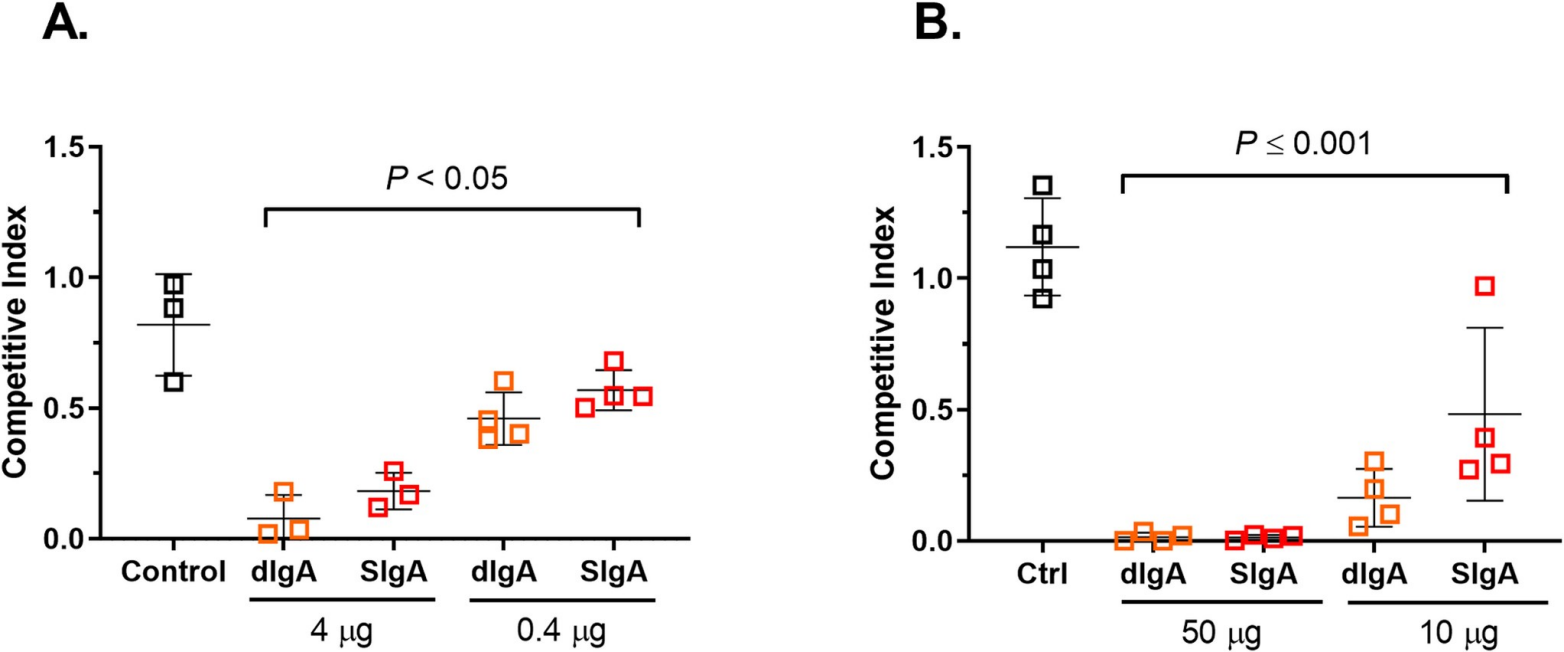
Comparison between Sal4 dimeric IgA (dIgA) and secretory IgA (SIgA) preparations in mouse model of STm infection. Adult BALB/c mice were challenged by gavage with a one-to-one mixture of STm O5 and O4 strains (4 x 10^7^ CFU total). Dimeric IgA (dIgA) or secretory IgA (SIgA) forms of Sal4 were (A) premixed with the bacterial inoculum or (B) administered ~1 min prior to STm challenge. The mice were euthanized 24 h later and Peyer’s patches were assessed for bacterial loads. Shown are the results of two independent experiments with 4 mice per group. Statistical significance evaluated for each concentration over the isotype control, as determined by one-way ANOVA and Tukey’s post-hoc test.

SC imparts a number of biologically important activities upon SIgA in the context of the intestinal lumen, including protease resistance and mucus affinity [11, 34]. We therefore expected that Sal4 SIgA would be significantly more effective *in vivo* than equivalent amounts of dimeric Sal4 IgA lacking SC. To test this hypothesis, purified, dimeric Sal4 (dIgA) and dIgA complexed with human recombinant SC (SIgA) were compared side-by-side in the mouse model of invasive STm. Analysis of bacterial burdens in intestinal tissues collected 24 h after challenge revealed that Sal4 dIgA and SIgA were equally effective at limiting uptake of AR05 into Peyer’s patches (**Fig 4A**), indicating that the addition of SC did not enhance the function of Sal4 dIgA in this model.

We postulated that the advantage of SC may only be apparent when antibody interacts with the intestinal environment in advance of bacterial challenge. We therefore repeated the experiments in which Sal4 dIgA and SIgA were given to mice by gavage immediately before STm challenge. Once again, however, Sal4 SIgA was no more effective than Sal4 dIgA at reducing invasion of AR05 into Peyer’s patch tissues. We conclude that, at least in this model of passive oral immunization, the potency of Sal4 IgA is not enhanced by the addition of SC (**Fig 4B**).

### Potential of orally administered Sal4 IgG to passively immunize mice

In clinical trials, ingestion of bovine milk- or colostrum-derived immunoglobulins consisting mainly of IgG from immunized dairy cows is sufficient to significantly reduce ETEC infection in adult volunteers [7, 35], indicating a role for IgG in passive oral immunizations. In fact, in a recent report, orally delivered anti-colonization factor antigen CFA/I IgG and SIgA human mAbs were equally effective at blocking ETEC infection in a mouse model [9].

**Fig 5:**
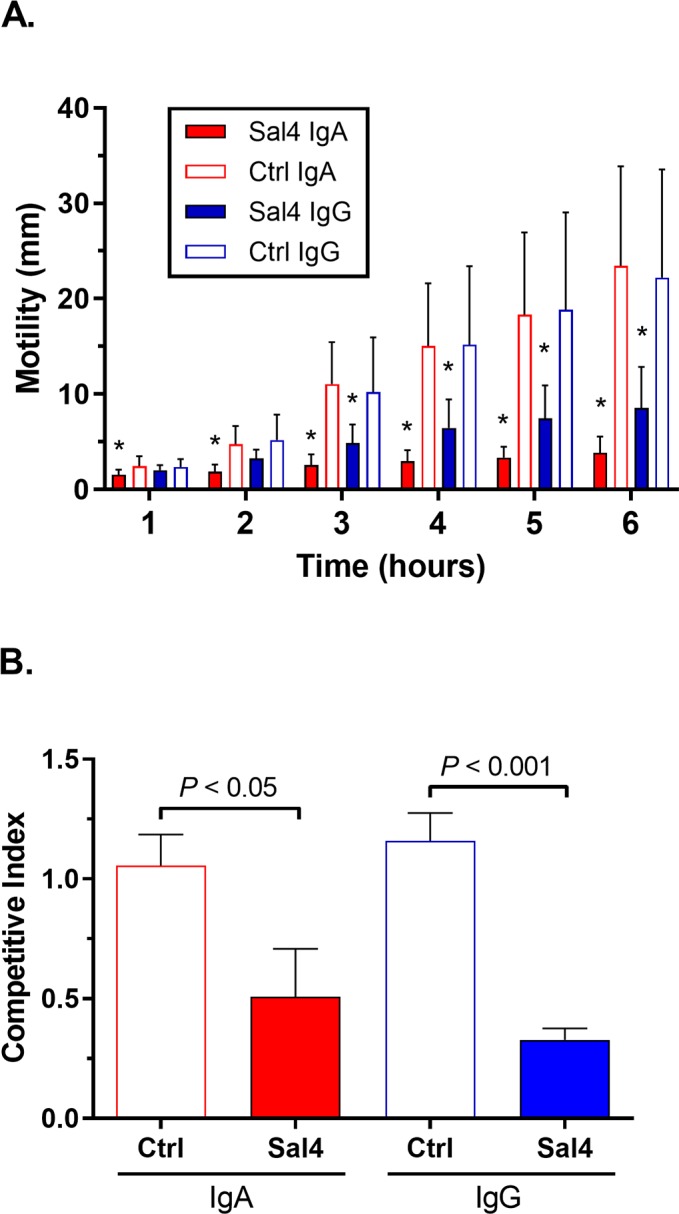
*In vitro* functionality of Sal4 IgG. (A) Effect of Sal4 IgG on STm motility in soft agar. STm was stab-inoculated into 0.3% LB agar containing 15 μg/mL of IgA control, Sal4 IgA, IgG control, or Sal4 IgG antibody. Plates were incubated at 37°C and the diameter of bacterial swimming was measured every hour for 6 h (*n* = 3 experiments each done in triplicate). (B) A one-to-one mixture of AR05 and AR04 STm (10^7^ CFUs total) were incubated for 10 min with 15 μg/mL of IgA control, Sal4 IgA, IgG control, or Sal4 IgG antibody before being applied to HeLa cell monolayers (MOI ~10), as described in the Materials and Methods. After 1 h, the HeLa cells were lysed and the CFUs were enumerated. Asterisks indicate significant reduction in wildtype STm motility (A) or invasion (B) over the respective isotype control, as determined by unpaired Student’s *t*-test (*n* = 2 experiments done in triplicate; *P* < 0.05).

To investigate the potential of orally administered IgG to prevent STm invasion of Peyer’s patch tissues, we engineered a Sal4 IgG chimeric antibody in which the V_H_ and V_L_ domains of the Sal4 IgA were grafted onto a human IgG1 framework (**S5 Fig**). The resulting Sal4 IgG1 was expressed in *Nicotiana benthamiana* using so-called RAMP technology [21]. The chimeric IgG1 mAb reacted with STm LPS by ELISA (**S5 Fig**) and promoted agglutination of STm in liquid culture. In a soft agar motility assay, Sal4 IgG limited bacterial spread over the course of the 6 h experiment, although slightly less effectively than Sal4 IgA. In the HeLa cell invasion assay, Sal4 IgG and Sal4 IgA were more or less equivalent in their abilities to block AR05 uptake (**Fig 5**). We therefore conclude that the Sal4 IgG1 molecule has expected the biological activities associated with Sal4 IgA, at least *in vitro*.

We next investigated the potential of chimeric Sal4 IgG1 to passively immunize mice against STm infection in both systemic and oral challenge models. For the systemic challenge model, groups of BALB/c mice were administered Sal4 IgG1 or a chimeric IgG1 isotype control (PB10) by intraperitoneal injection and then challenged 24 h later with 10^4^ CFUs of wild type STm ATCC14028 by the same route. One day later, the mice were euthanized and CFUs in the spleens and livers were evaluated. As compared to the IgG1 control group, mice that received the high dose Sal4 IgG1 (40 μg) had 10 to 100-fold lower STm burden in the spleens and livers (**S6 Fig**). Bacterial numbers were also reduced in mice that received a low dose (10 μg) of Sal4 IgG1, although to a lesser extent than the high dose group of animals. These results demonstrate that passively administered Sal4 IgG1 results in dose-dependent reduction in STm systemic infection.

**Fig 6:**
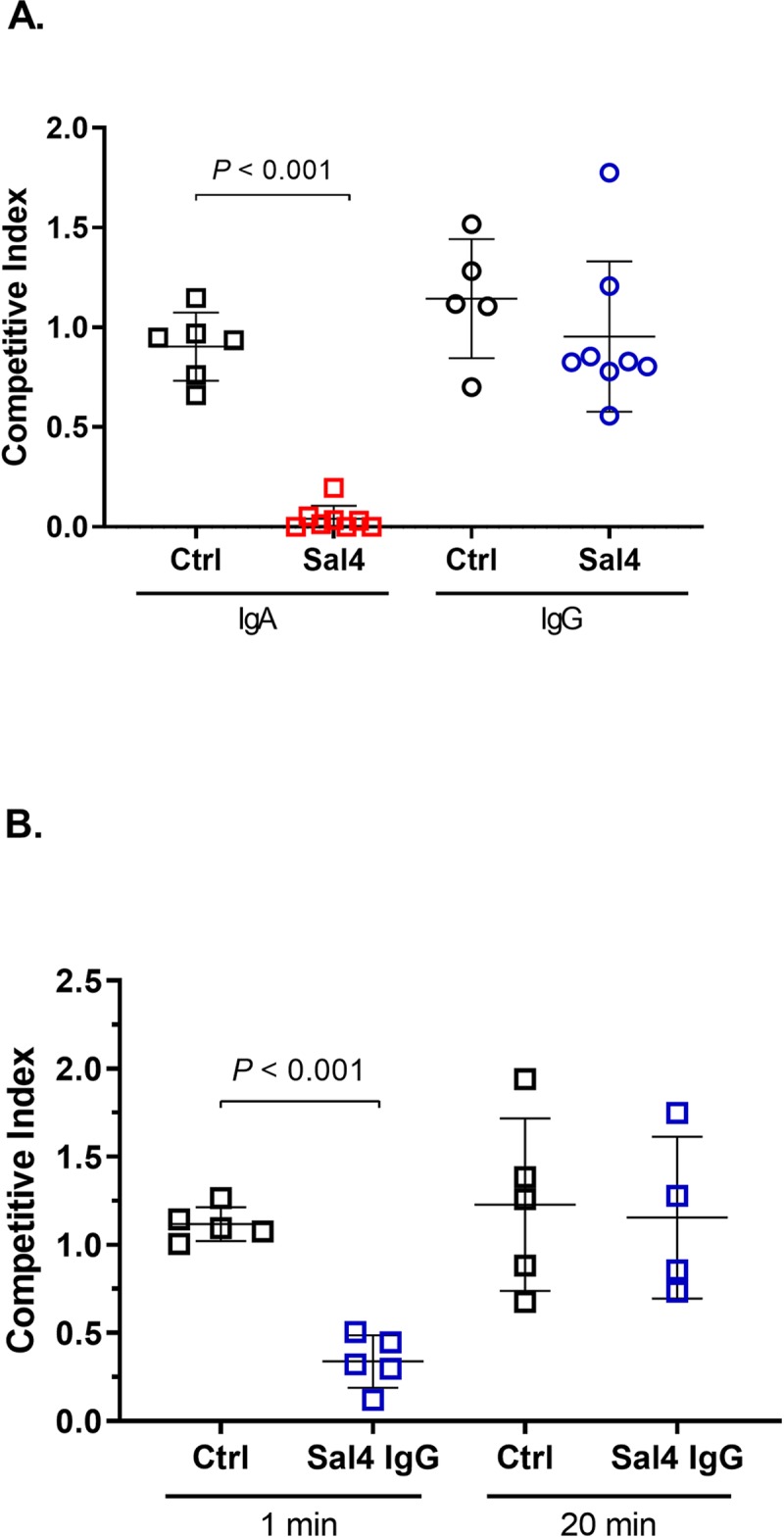
Sal4 IgG fails to inhibit STm invasion following oral infection *in vivo*, but can be partially rescued upon stabilization. BALB/c females were orally challenged with a competitive index of wildtype (AR05) and *oafA* mutant (AR04) STm (4 x 10^7^ CFUs) either (A) pre-incubated with 30 μg/mL of antibody in PBS or (B) administered antibody prior to STm challenge at the indicated time points with sodium bicarbonate and protease inhibitors. 24 hours (p.i.) mice were euthanized and Peyer’s patches were harvested and enumerated for CFUs and competitive indices. Statistical significance evaluated for each group over the isotype control, as determined by unpaired Student’s *t*-test (*n* = at least 5 mice per group).

To examine Sal4 IgG1’s activity in the context of intestinal immunity to STm, groups of BALB/c mice were gavaged with a 1:1 mixture of AR05 and AR04 supplemented with 30 μg/mL Sal4 IgA or IgG1 or the relevant isotype controls. Invasion of Peyer’s patch tissues was measured 24 h later. As observed previously, Sal4 IgA reduced AR05 invasion into Peyer’s patch tissues by several orders of magnitude (CI value of 0.04 ± 0.02) (**Fig 6**). In contrast, Sal4 IgG1 had no effect on STm invasion, as evidenced by a CI value of 0.95 ± 0.13 (**Fig 6**). The failure of Sal4 IgG1 to function in these studies could not be overcome by increasing antibody dose (e.g., >750 μg) or repeated administration over a 12 h period (**S7 Fig**) or by high dose parenteral administration (**S8 Fig**).

We postulated that antibody stability in the gastric environment might account (at least in part) for the failure of Sal4 IgG1 to function in the oral passive immunization model [11, 24].To address this experimentally, Sal4 IgA and IgG1 variants were incubated in an adult simplified Simulated Gastric Fluid (SGF), without and with pepsin, essentially as described [22, 23]. After 10, 30, and 60 min at 37°C, aliquots were removed and tested reactivity with STm LPS by ELISA. In the presence of SGF, Sal4 IgG1 levels declined steadily over a 30 min time period, whereas the IgA1 variant was relatively stable (**S1 Table**). Upon the addition of pepsin, however, IgG1 declined so precipitously that it was undetectable at 10 min. At the same time point (10 min), Sal4 IgA had declined to just ~5% of starting levels, but then remained detectable until 30 min. Collectively, these results confirmed the differential sensitivity of Sal4 IgG1 and IgA to the gastric environment.

The in vitro stability studies with Sal4 IgG prompted us to repeat the passive immunization studies with the addition of sodium bicarbonate plus protease inhibitors. Specifically, Sal4 IgG1 in sodium bicarbonate (3% NaHCO_3_) plus protease inhibitors administered to mice by gavage 1 min or 20 min prior STm challenge. Under these conditions, Sal4 IgG1 did in fact block STm invasion into Peyer’s patch tissues, but only when given immediately before STm challenge (**Fig 6**). Collectively, these results suggest that ineffectiveness of Sal4 IgG1, as compared to Sal4 IgA, is due to its instability in the gut environment.

## Discussion

In this study, we investigated the potential of orally administered mAbs to passively immunize mice against invasive Salmonella. The study was motivated by several factors. First is the rapid emergence of multi-drug resistance *Salmonella* infections, which constitute an increasing threat to public health in developing and developed countries [36, 37]. Second, given the difficulty and extended timeline associated with vaccine development, there is pressure from federal and private foundations such as the Bill and Melinda Gates Foundation to explore alternative strategies as a means of protecting at risk individuals from debilitating enteric infections. With the remarkable advances in recombinant mAb engineering and scale-up using mammalian cells, transgenic animals, plants and even seed-based production platforms, the prospect of combatting diarrheal diseases through orally administered mAb cocktails is technically feasible and cost-effective [33, 38, 39].

We found that direct administration of Sal4 IgA to adult mice by gavage overcomes many of the impediments associated with the so-called “backpack” tumor model that was used previously to study STm-IgA interactions [29]. In the backpack model, antibody-secreting B cell hybridomas are implanted subcutaneously into mice, resulting in local tumor formation and the accumulation of antibodies at very high concentrations in serum and interstitial fluids, including the lamina propria [13]. Ultimately, hybridoma-derived, antigen-specific IgA is detected in intestinal secretions, presumably as a result of pIgR-mediated transcytosis [40, 41]. While this set-up resulted in physiologic delivery of Sal4 IgA into the intestinal lumen, the are several drawbacks with the model. First, depending on how well the hybridoma “takes,” the amount of Sal4 IgA in serum and intestinal secretions varies widely from mouse to mouse, thereby confounding the ability to perform strict dose-response studies. Second, because hybridoma-derived antibodies accumulate at potentially very high levels in serum (1–10 mg/ml) and interstitial fluids, it is not always possible to delineate whether observed protection is due to intestinal (secretory) or interstitial antibodies [40]. Finally, while it is assumed Sal4 IgA antibodies detected in the intestinal secretions in the backpack tumor mice are complexed with SC, the actual amount of Sal4 SIgA in the lumen has never been determined. Direct administration of Sal4 IgA of known molecular forms and at specific doses overcomes these concerns.

The other notable benefit of the challenge model employed here is that the primary readout is bacterial load (CFUs) in Peyer’s patch tissues, which are known to be the primary point of entry for invasive *Salmonella* [42, 43]. Uptake into Peyer’s patches occurs through M cells and is dependent on the SPI-1 T3SS. Moreover, Peyer’s patch invasion occurs in the presence of a normal gut microbiota. This is in contrast to models of *Salmonella*-induced inflammation where infection occurs primarily in the cecum and colon and involves pre-treatment of mice with antibiotics like streptomycin to deplete the gut microbiota [44, 45]. For the purposes of this study, the challenge model granted us the ability to ask vital questions about IgA biology, such as the importance of SC in intestinal immunity.

Indeed, contrary to what we expected, the addition of SC did not augment Sal4 IgA activity in our mouse model of invasive STm. One possible explanation for this finding relates to the route of antibody delivery. Normally, dIgA is transported across the intestinal epithelium by the pIgR, which is preferentially expressed by enterocytes in intestinal crypts. Following transport, SIgA localizes to the mucus layer overlying the epithelial barrier where SC plays a central role in anchoring IgA within this microenvironment and protecting the antibody from protease-mediated degradation [20, 46, 47]. It is unclear if the physiologic distribution of SIgA is recapitulated when antibody is administered by gavage. Our attempts to track Sal4 SIgA, using immunohistochemistry, in the small intestine following oral delivery have not been successful to date. Another possible explanation for why SC did not impart a benefit to Sal4 IgA is that the rate-limiting determinant for antibody activity in this model is dilution effects upon gavage, not protease sensitivity or mucus anchoring, where SC would be expected to play an important role.

The comparison between PeA3 and Sal4 IgA mAbs in the mouse model of invasive Salmonella serves as an indirect demonstration of the importance of IgA avidity in protecting against invasive pathogens. By all accounts, Sal4 and PeA3 recognized the same or a very similar epitope, but differed in their relative binding affinities for the O5 antigen by ~10 fold. The difference in binding activity correlated with differences in *in vivo* efficacy, at least when PeA3 and Sal4 IgA were given to mice at lower doses. At higher doses, Sal4 and PeA3 were equally effective at limiting STm uptake into Peyer’s patch tissues, underscoring that protection of the mucosal surface is due to the interrelationship between IgA avidity and local antibody concentrations. As illustrated by Cothésy et al., even polyreactive SIgA is protective if present at sufficiently high concentrations [11]. From the standpoint of passive immunization, however, higher affinity/avidity antibodies are clearly advantageous since much lower doses would be required to achieve protection. Indeed, in the case of respiratory infections, the selection for higher affinity mAbs resulted in correspondingly higher neutralizing activities and in *vivo* potency [48].

We found that a recombinant human IgG1 variant of Sal4 had only marginal capacity (when co-administered with protease inhibitors and sodium bicarbonate) to passively immunize mice against intragastric Salmonella infection. These findings are consistent with IgG1 instability in the gastric environment. It is likely that the heavily glycosylated nature of IgA provides an advantage upon direct delivery into the gut in terms of maintaining both direct antigen binding and crosslinking between multiple antigens [46], while the IgG mAb, with a lone pair of N-glycans on the Fc region [49], is outmatched. Other factors may also be at play. Sal4 IgG1, which is a monomer, likely differs from Sal4 IgA, which is a dimer, in its ability to promote bacterial agglutination. We cannot rule out the possibility that the nature of agglutination between IgG and IgA is quantitatively different considering that we did observe slight differences in the kinetics of microagglutination between to two antibody isotypes (**S2 Table**).

Ultimately, our study highlights some of the fundamental challenges associated with oral mAb delivery as a means to combat enteric diseases, especially in children. Foremost is the remarkably short apparent “stability” of Sal4 IgA and Sal4 SIgA in the gastric and intestinal environments. As noted above, our results suggest that the rapid decline of Sal4 IgA activity when given prophylactically to mice is likely due to a combination of physical degradation by local proteases and acid pH, coupled with rapid clearance/dilution in the intestinal lumen. Even modest declines in the local concentration of Sal4 IgA would be expected to impact antibody potency, considering that the ability of Sal4 IgA to block bacterial entry into Peyer’s patches is the result (at least in part) of bacterial agglutination in the intestinal lumen. For Sal4 or other IgA mAbs to be used clinically in the future will require a more thorough understanding of the pharmacokinetics of IgA and SIgA in mucosal tissues. Coupled with those efforts is a need to identify formulations and delivery strategies to ensure proper delivery and localization of IgA and SIgA in the regions of the gut where they are most needed. In the case of STm, that would be in the proximal small intestine, while for Shigella species the antibodies would need to reach the colon to exert their effects. Nonetheless, in spite of these barriers, specific oral IgG preparations from HBC have seen success therapeutically in clinical trials of children with rotavirus diarrhea [50], prophylactically in rotavirus-infected mice [51] and ETEC challenged humans [52, 53], suggesting the goal of using passive immunization to combat enteric disease is not far from reach.

## Supporting information

S1 Table Differences in antibody stability during *in vitro* digestion between Sal4 IgA and IgG. Sal4 IgA and IgG (100 μg/mL each) were incubated at 37°C in adult simulated gastric fluid (SGF) with or without the addition of pepsin (2000 U/mL) at pH 3.0. Aliquots were then taken after incubating for 10, 30, and 60 minutes and adjusted to a pH of 7.0–7.4 using 1M NaOH. Neutralized samples were assessed for STm-LPS binding by ELISA and the amount of Sal4 IgA and IgG remaining was calculated using a standard curve with SoftMax Pro 5.2 software.
(TIF)

S2 Table STm agglutination kinetics by Sal4 IgA and IgG. Overnight liquid STm cultures (ATCC 14028) were grown and adjusted to an OD_600_ of 0.7 and incubated with 1, 3, 10, or 30 μg/mL of Sal4 IgA or IgG at 37°C at the indicated time points under static conditions. (+) indicates the presence of macro-agglutination, while (-) indicates the absence of agglutination at a given time point and concentration.
(TIF)

S1 Fig Sal4 IgA binding to AR04 and AR05 by whole-bacteria ELISA. Sal4 IgA reactivity to STm strains AR04 and AR05 by whole-cell ELISA, as described in the Materials and Methods.
(TIF)

S2 Fig Schematic of workflow for STm intragastric challenge model. (1) A 1:1 mixture of wildtype (AR05) and mutant (AR04) STm is incubated with antibody for 10 minutes. (2) BALB/c mice are challenged with antibody-treated STm inoculum (4 x 10^7^ CFUs total per mouse). (3) 24 h post-infection mice are euthanized and Peyer’s patches from each mouse are collected and homogenized. (4) Tissue homogenates are plated on LB agar containing kanamycin (50 μg/mL) and X-Gal (40 μg/mL) to evaluate antibody-dependent changes on STm infection by blue-white screening. Images generated using Microsoft Office suite.
(TIF)

S3 Fig PeA3 IgA *in vitro* characterization. (A) Sal4 IgA and PeA3 IgA reactivity to STm purified LPS (Sigma), STm strains CS022 and SJF59, and *S*. Enteritidis by ELISA. (+) indicates positive binding, while (-) indicates no binding detected above background levels. (B) Agglutination of AR05 liquid culture by 15 μg/mL of Sal4 IgA and PeA3 IgA after incubation at 37°C for 60 minutes. (C) Effect of Sal4 IgA and PeA3 IgA (15 μg/mL) on STm motility in 0.3% soft agar. Plates were incubated at 37°C and the diameter of bacterial swimming was measured every hour for 6 hours. Asterisks indicate significant reduction in wildtype STm motility over the isotype control, as determined by Kruskal-Wallis and Dunn’s multiple comparisons tests at each time point (P < 0.05). (D) Binding of Sal4 IgA and PeA3 IgA to purified STm LPS by ELISA. For additional experimental details see Materials and Methods.
(TIF)

S4 Fig Sal4 IgA prophylactic activity in PBS. BALB/c mice were passively immunized orally with either control (2D6 IgA) or Sal4 IgA antibody treatment in PBS at the indicated doses. 20 minutes later mice were challenged with a 1:1 mixture of STm strains AR04 and AR05 (4 x 10^7^ CFUs/mouse). 24 h post-infection Peyer’s patches were harvested and enumerated for CIs, as described in the Materials and Methods.
(TIF)

S5 Fig Sal4 IgG binds to STm O5-Antigen and competes with Sal4 IgA. (A) Sal4 IgG reacts to STm strain AR05, but not AR04, as determined by whole-cell ELISA (described in the Materials and Methods). (B) Sal4 IgG binding to purified STm LPS by ELISA and Sal4 IgG competition ELISA with Sal4 IgA. Sal4 IgA at the indicated concentrations was applied to purified STm LPS-coated plates and incubated for 1 h at room temperature. Plates were washed three times with PBST, and 10 μg/mL of Sal4 IgG was applied and incubated for an additional hour. Plates were then developed using goat anti-human HRP-conjugated secondary IgG antibody and SureBlue TMB Microwell Peroxidase Substrate to evaluate Sal4 IgG inhibition by Sal4 IgA.
(TIF)

S6 Fig Sal4 IgG and IgA significantly reduce bacterial burden in systemic organs following systemic STm challenge. BALB/c mice were passively immunized with (A and B) Sal4 IgG or (C and D) Sal4 IgA at the indicated doses by intraperitoneal injection 24 h prior to a systemic lethal challenge of STm (1 x 10^4^ CFUs). Control mice received isotype control-matched mAbs (PB10 IgG, 2D6 IgA) as described in the Materials and Methods. For technical reasons, the 2D6 IgA treatment group received only 21 μg as opposed to 40 μg. 24 h post-infection, mice were euthanized, and the spleens and livers were harvested, homogenized, and plated for CFUs on LB agar. Asterisks indicate significant reduction bacterial burden compared to isotype control treatment as determined by one-way ANOVA and Tukey’s post-hoc test; *P < 0.05, **P < 0.01, ****P < 0.0001.
(TIF)

S7 Fig Sal4 IgG does not significantly block invasion *in vivo* after multiple administrations. BALB/c mice were orally administered 190 μg of isotype control (PB10 IgG) or Sal4 IgG antibody treatment in PBS in multiple doses at 2.5 h and 20 min before STm challenge (4 x 10^7^ CFUs of AR04 and AR05) and 15 min and 4.5 h following challenge for a total dose of 760 μg per mouse. 24 h post-infection Peyer’s patches were harvested and enumerated for CFUs and CIs as described in the Materials and Methods. No statistical significance between the control and Sal4 IgG treatment groups was observed, as determined by unpaired Student’s *t*-test (*P* = 0.35).
(TIF)

S8 Fig Intraperitoneal administration of Sal4 IgG does not significantly block invasion *in vivo* after oral STm challenge. 200 μg of either Sal4 IgG or isotype control antibody (PB10 IgG) was administered via intraperitoneal injection. This corresponded to an average of 39.9 μg/mL (± SD of 5.8) of Sal4 IgG present in the serum of mice at the time of challenge as determined by ELISA (samples from *n* = 3 mice). 24 h after antibody administration, mice were challenged orally with STm inoculum containing a 1:1 mixture of strains AR04 (mutant) and AR05 (wildtype). 24 h post-infection, mice were euthanized, and Peyer’s patches harvested and enumerated for CIs, as described in the Materials & Methods section. No statistical significance between the control and Sal4 IgG treatment groups was observed, as determined by unpaired Student’s *t*-test (*P* = 0.74).
(TIF)
