# Supplementary figures and images for: Inhibition of invasive salmonella by orally administered IgA and IgG monoclonal antibodies
Source: PLoS Negl Trop Dis. 2020 Mar 23;14(3):e0007803. doi: 10.1371/journal.pntd.0007803 (PMC7117778; doi:10.1371/journal.pntd.0007803)

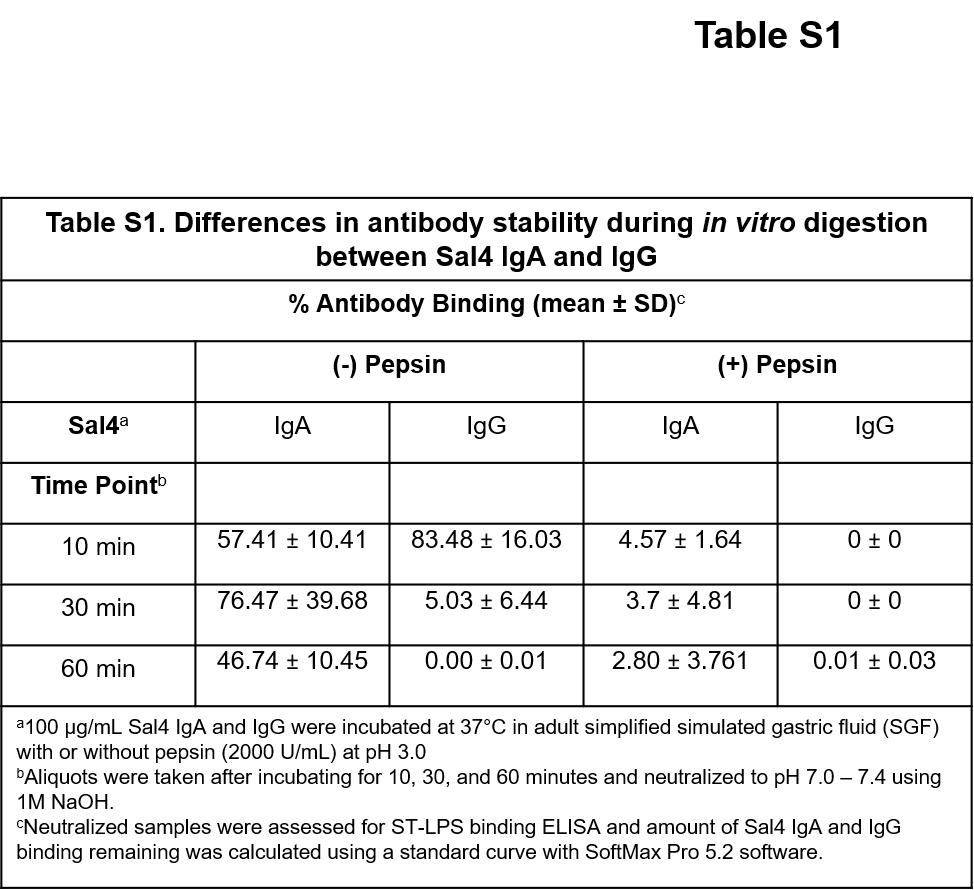

Supplement: S1 Table — Sal4 IgA and IgG (100 μg/mL each) were incubated at 37°C in adult simulated gastric fluid (SGF) with or without the addition of pepsin (2000 U/mL) at pH 3.0. Aliquots were then taken after incubating for 10, 30, and 60 minutes and adjusted to a pH of 7.0–7.4 using 1M NaOH. Neutralized samples were assessed for STm-LPS binding by ELISA and the amount of Sal4 IgA and IgG remaining was calculated using a standard curve with SoftMax Pro 5.2 software. (TIF) [file pntd.0007803.s001.tif]

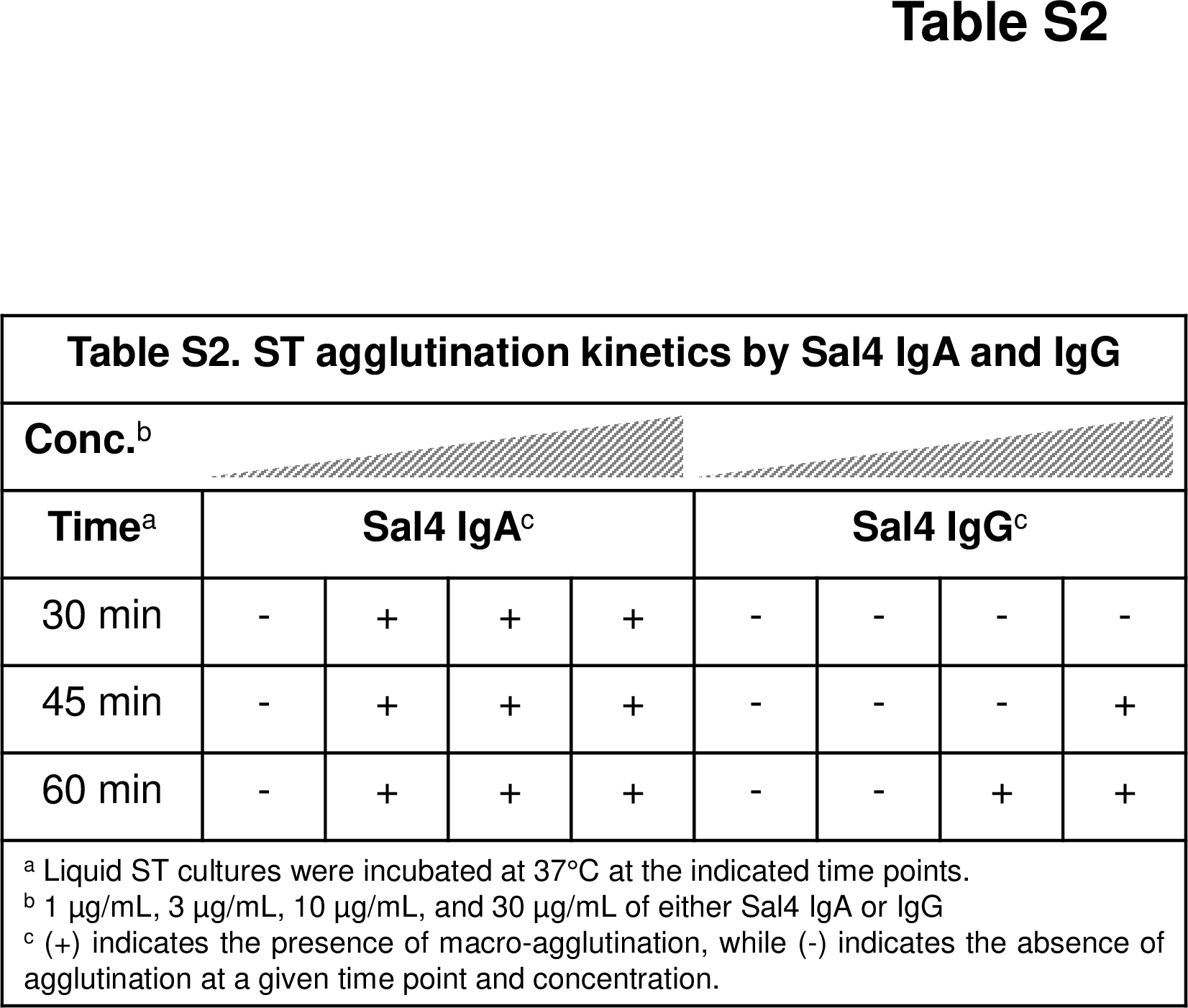

Supplement: S2 Table — Overnight liquid STm cultures (ATCC 14028) were grown and adjusted to an OD600 of 0.7 and incubated with 1, 3, 10, or 30 μg/mL of Sal4 IgA or IgG at 37°C at the indicated time points under static conditions. (+) indicates the presence of macro-agglutination, while (-) indicates the absence of agglutination at a given time point and concentration. (TIF) [file pntd.0007803.s002.tif]

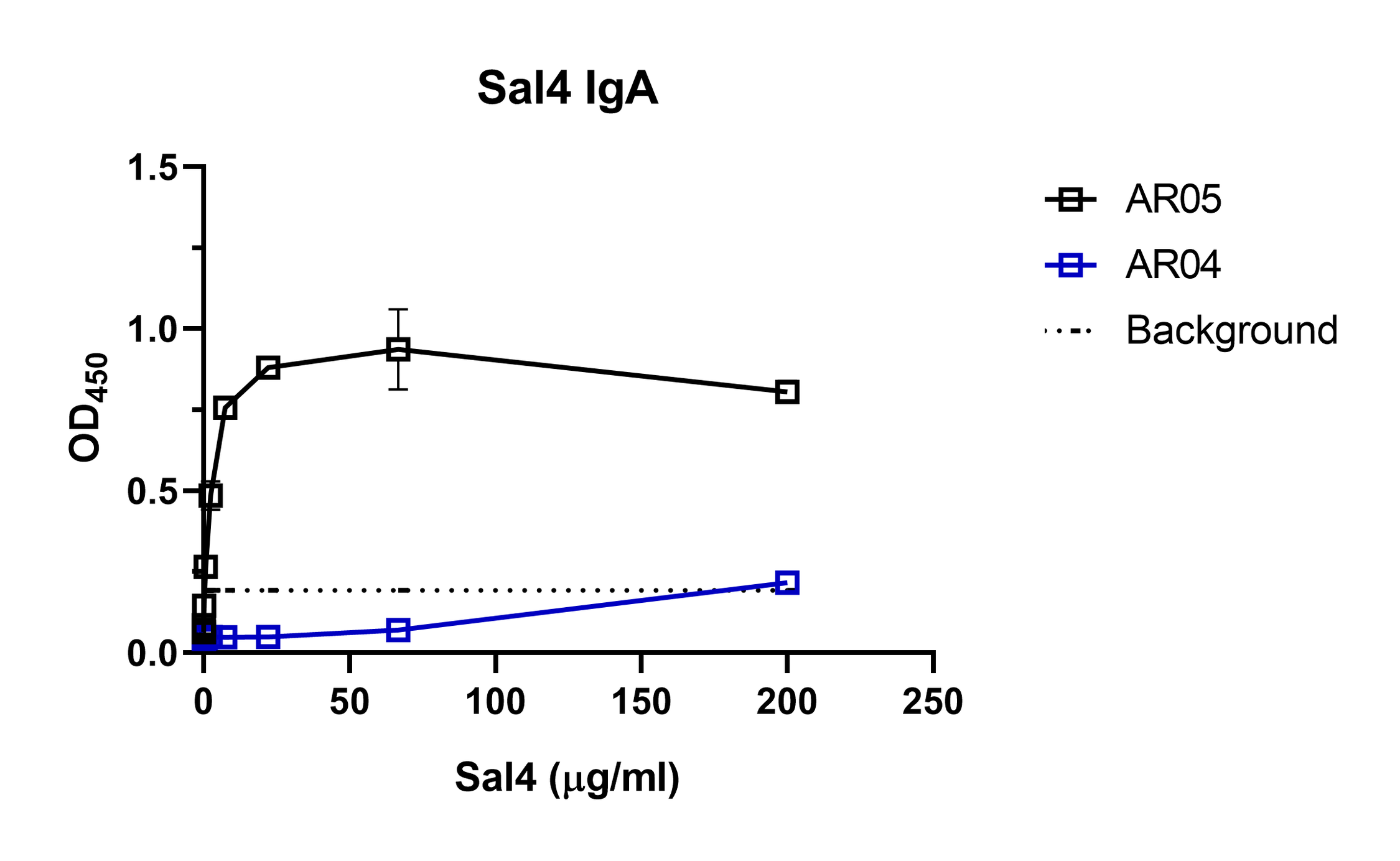

Supplement: S1 Fig — Sal4 IgA reactivity to STm strains AR04 and AR05 by whole-cell ELISA, as described in the Materials and Methods. (TIF) [file pntd.0007803.s003.tif]

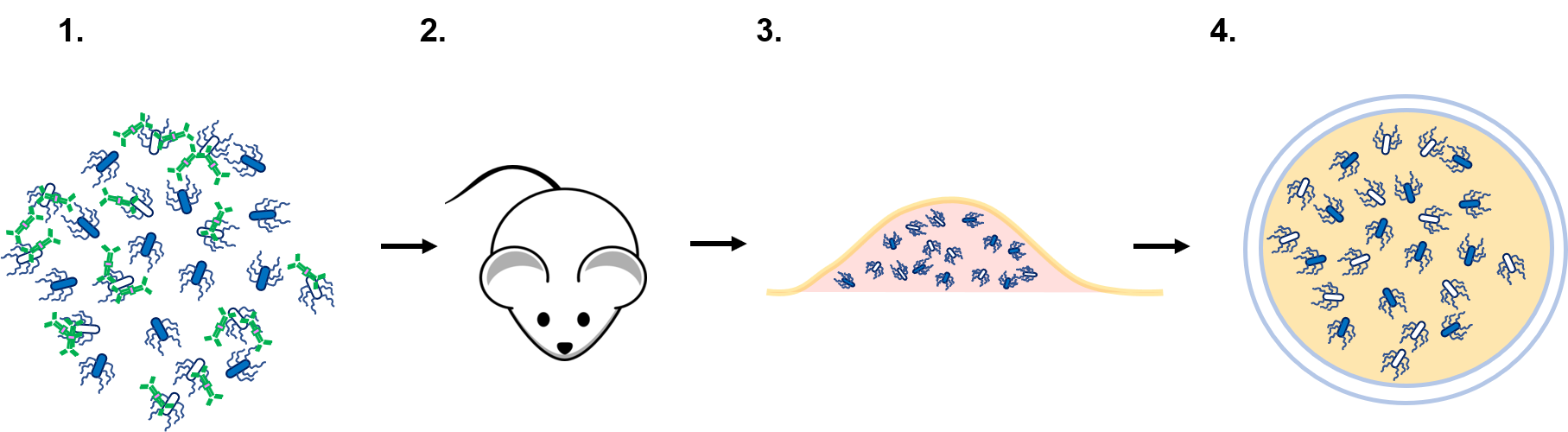

Supplement: S2 Fig — (1) A 1:1 mixture of wildtype (AR05) and mutant (AR04) STm is incubated with antibody for 10 minutes. (2) BALB/c mice are challenged with antibody-treated STm inoculum (4 x 10^7^ CFUs total per mouse). (3) 24 h post-infection mice are euthanized and Peyer’s patches from each mouse are collected and homogenized. (4) Tissue homogenates are plated on LB agar containing kanamycin (50 μg/mL) and X-Gal (40 μg/mL) to evaluate antibody-dependent changes on STm infection by blue-white screening. Images generated using Microsoft Office suite. (TIF) [file pntd.0007803.s004.tif]

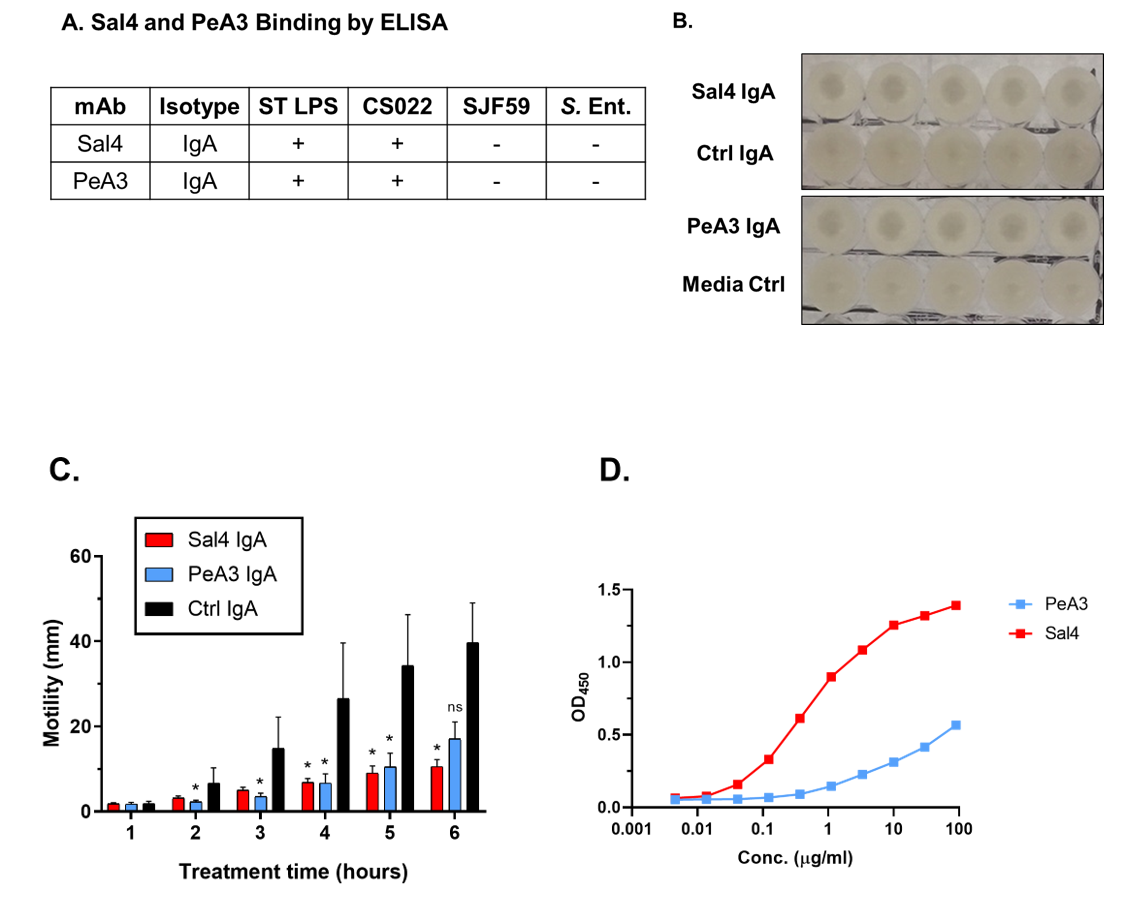

Supplement: S3 Fig — (A) Sal4 IgA and PeA3 IgA reactivity to STm purified LPS (Sigma), STm strains CS022 and SJF59, and S. Enteritidis by ELISA. (+) indicates positive binding, while (-) indicates no binding detected above background levels. (B) Agglutination of AR05 liquid culture by 15 μg/mL of Sal4 IgA and PeA3 IgA after incubation at 37°C for 60 minutes. (C) Effect of Sal4 IgA and PeA3 IgA (15 μg/mL) on STm motility in 0.3% soft agar. Plates were incubated at 37°C and the diameter of bacterial swimming was measured every hour for 6 hours. Asterisks indicate significant reduction in wildtype STm motility over the isotype control, as determined by Kruskal-Wallis and Dunn’s multiple comparisons tests at each time point (P < 0.05). (D) Binding of Sal4 IgA and PeA3 IgA to purified STm LPS by ELISA. For additional experimental details see Materials and Methods. (TIF) [file pntd.0007803.s005.tif]

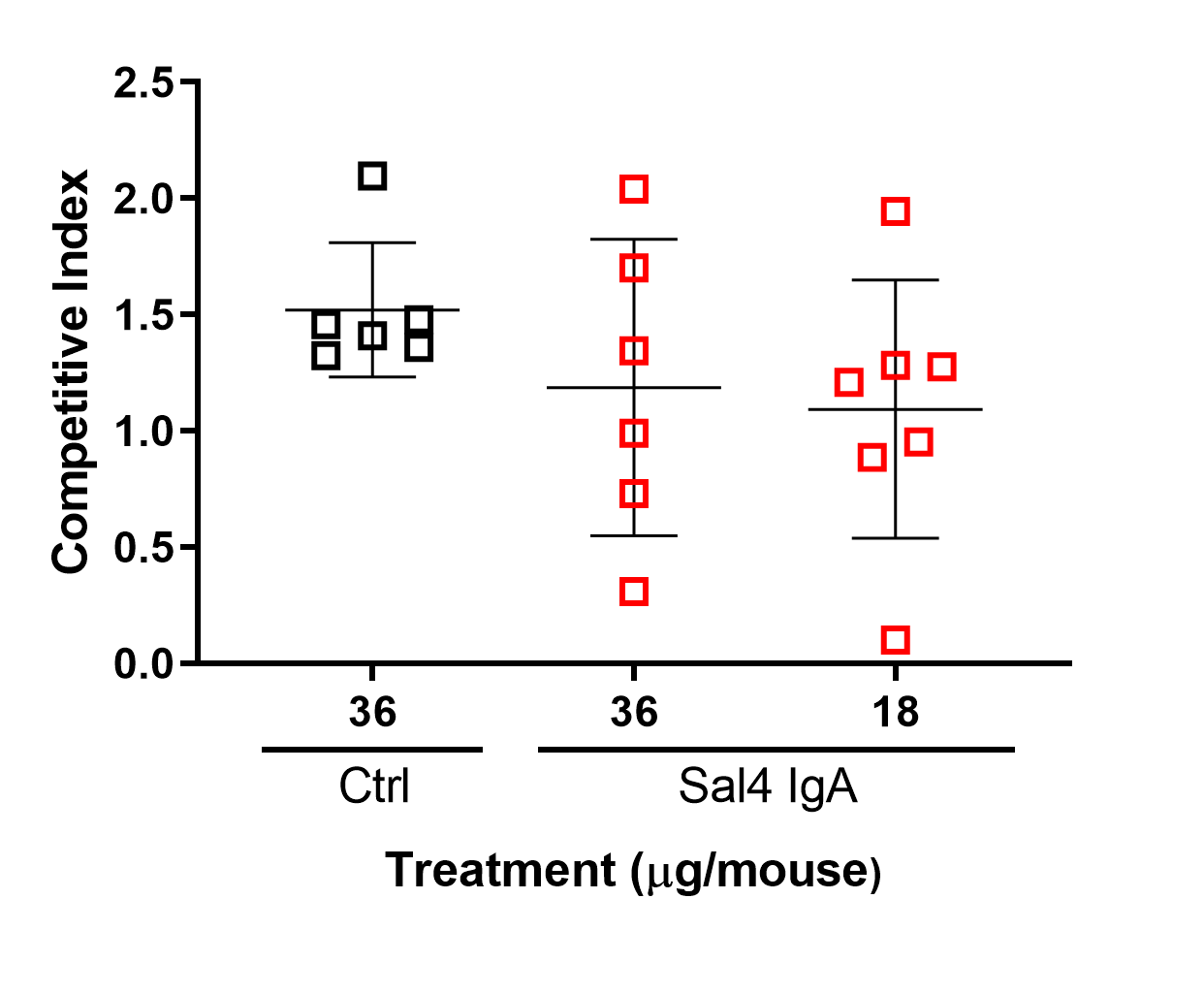

Supplement: S4 Fig — BALB/c mice were passively immunized orally with either control (2D6 IgA) or Sal4 IgA antibody treatment in PBS at the indicated doses. 20 minutes later mice were challenged with a 1:1 mixture of STm strains AR04 and AR05 (4 x 10^7^ CFUs/mouse). 24 h post-infection Peyer’s patches were harvested and enumerated for CIs, as described in the Materials and Methods. (TIF) [file pntd.0007803.s006.tif]

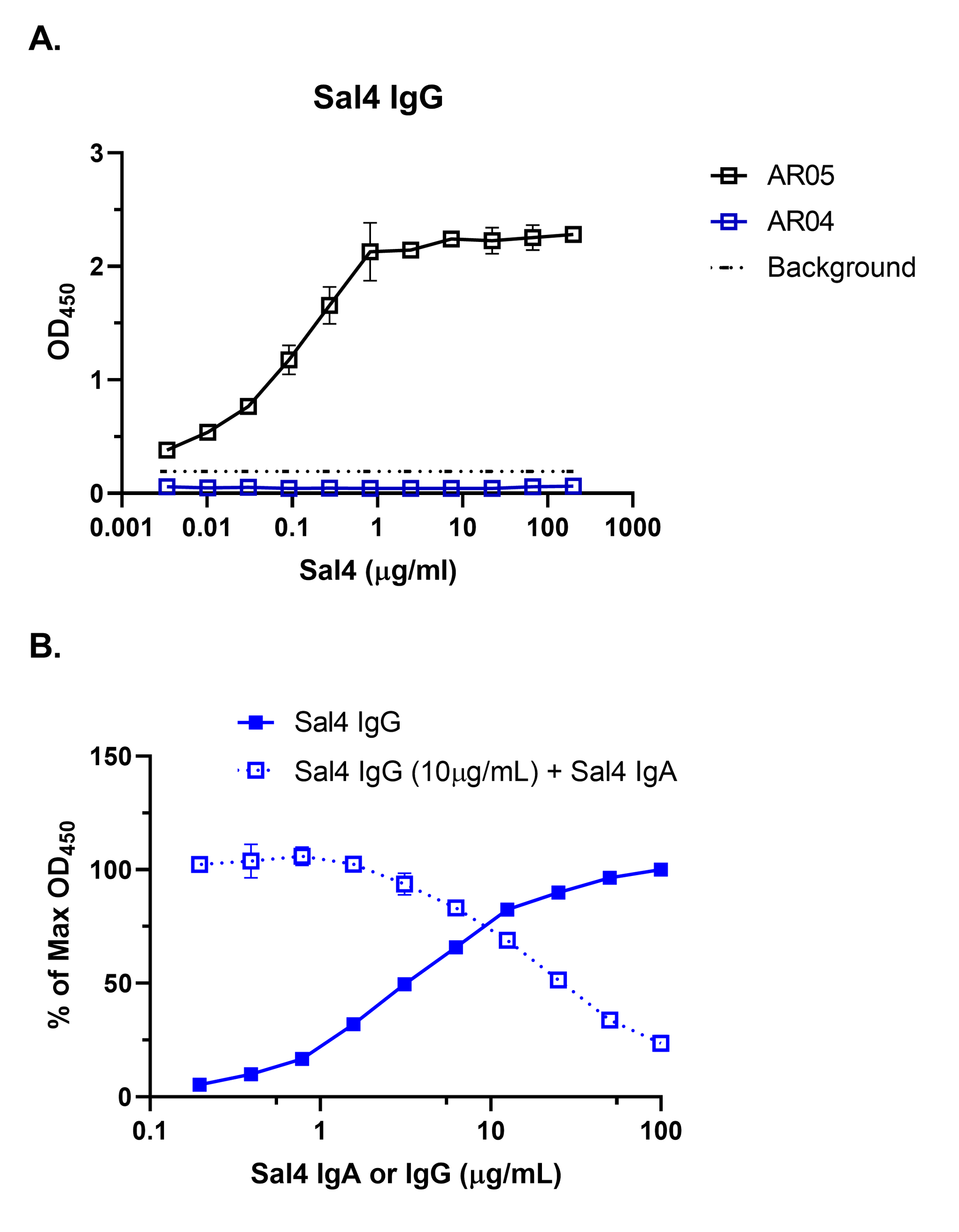

Supplement: S5 Fig — (A) Sal4 IgG reacts to STm strain AR05, but not AR04, as determined by whole-cell ELISA (described in the Materials and Methods). (B) Sal4 IgG binding to purified STm LPS by ELISA and Sal4 IgG competition ELISA with Sal4 IgA. Sal4 IgA at the indicated concentrations was applied to purified STm LPS-coated plates and incubated for 1 h at room temperature. Plates were washed three times with PBST, and 10 μg/mL of Sal4 IgG was applied and incubated for an additional hour. Plates were then developed using goat anti-human HRP-conjugated secondary IgG antibody and SureBlue TMB Microwell Peroxidase Substrate to evaluate Sal4 IgG inhibition by Sal4 IgA. (TIF) [file pntd.0007803.s007.tif]

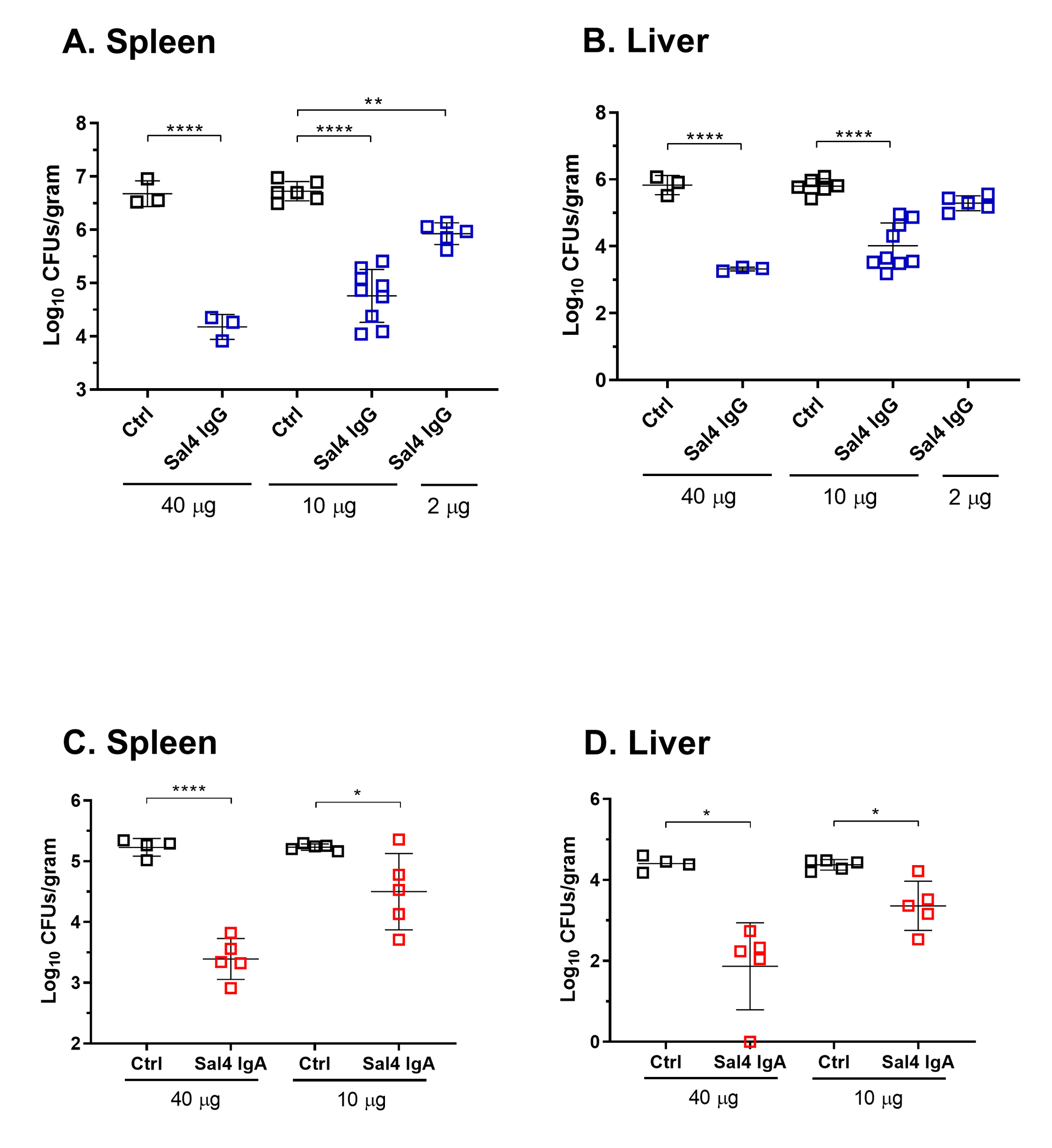

Supplement: S6 Fig — BALB/c mice were passively immunized with (A and B) Sal4 IgG or (C and D) Sal4 IgA at the indicated doses by intraperitoneal injection 24 h prior to a systemic lethal challenge of STm (1 x 10^4^ CFUs). Control mice received isotype control-matched mAbs (PB10 IgG, 2D6 IgA) as described in the Materials and Methods. For technical reasons, the 2D6 IgA treatment group received only 21 μg as opposed to 40 μg. 24 h post-infection, mice were euthanized, and the spleens and livers were harvested, homogenized, and plated for CFUs on LB agar. Asterisks indicate significant reduction bacterial burden compared to isotype control treatment as determined by one-way ANOVA and Tukey’s post-hoc test; *P < 0.05, **P < 0.01, ****P < 0.0001. (TIF) [file pntd.0007803.s008.tif]

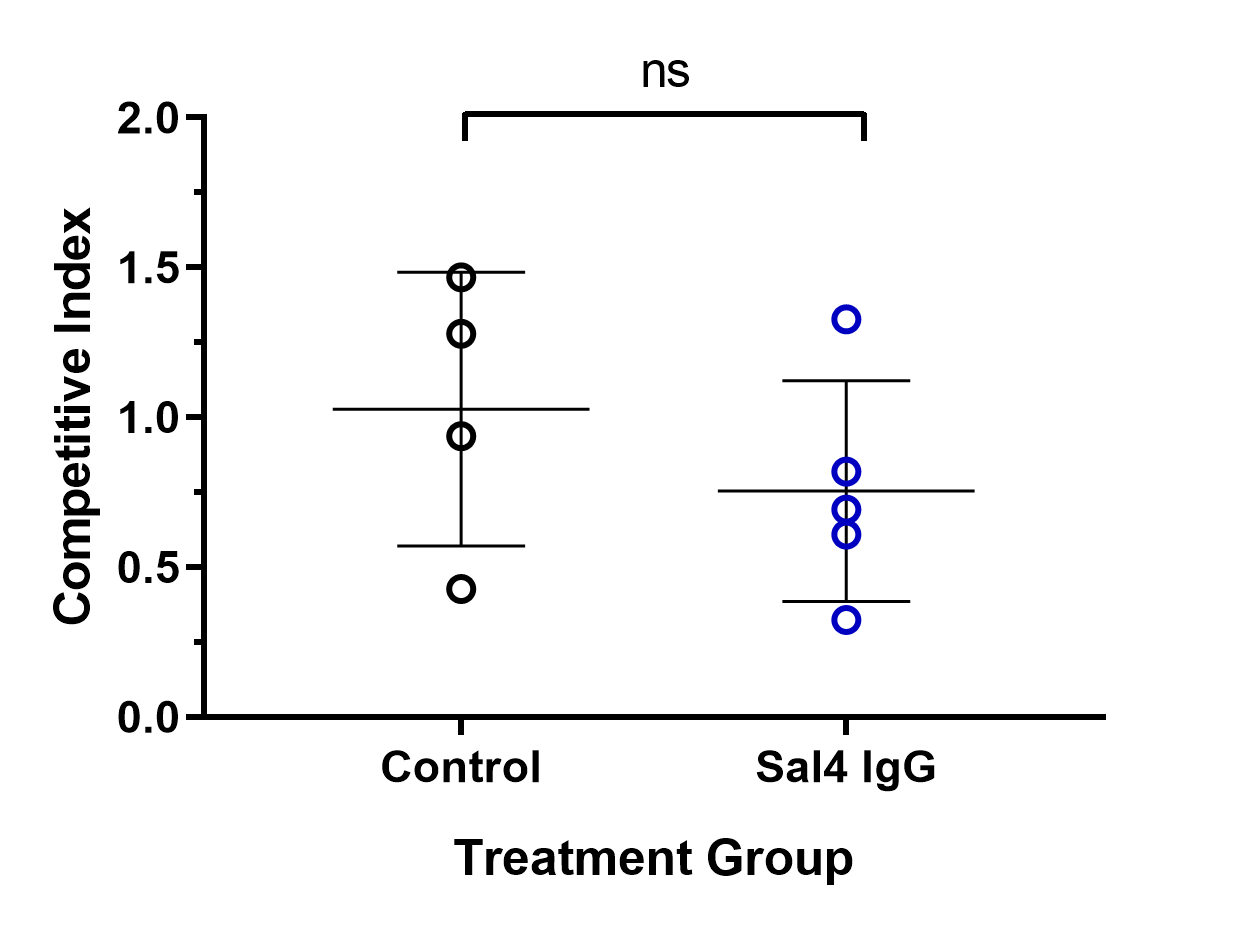

Supplement: S7 Fig — BALB/c mice were orally administered 190 μg of isotype control (PB10 IgG) or Sal4 IgG antibody treatment in PBS in multiple doses at 2.5 h and 20 min before STm challenge (4 x 10^7^ CFUs of AR04 and AR05) and 15 min and 4.5 h following challenge for a total dose of 760 μg per mouse. 24 h post-infection Peyer’s patches were harvested and enumerated for CFUs and CIs as described in the Materials and Methods. No statistical significance between the control and Sal4 IgG treatment groups was observed, as determined by unpaired Student’s t-test (P = 0.35). (TIF) [file pntd.0007803.s009.tif]

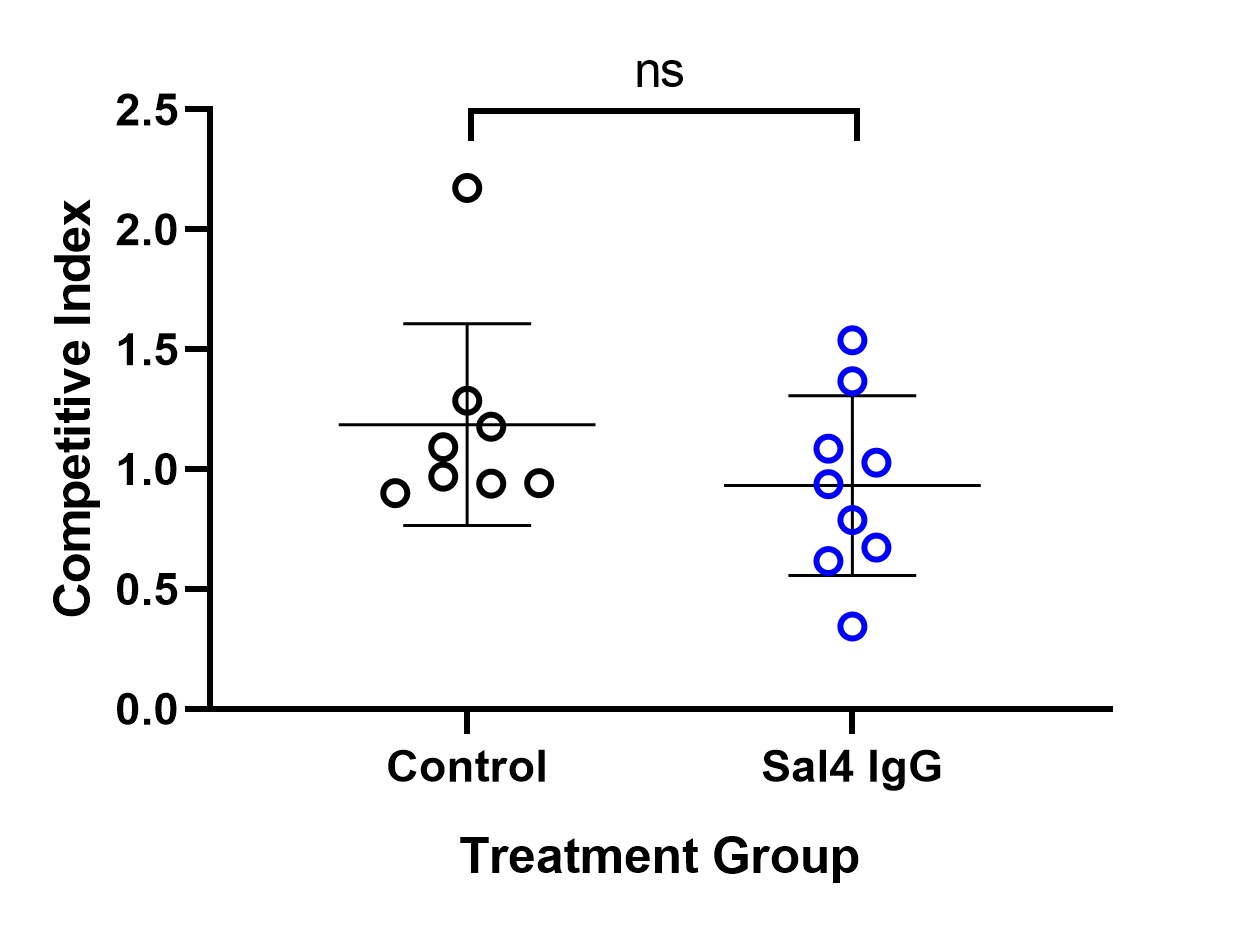

Supplement: S8 Fig — 200 μg of either Sal4 IgG or isotype control antibody (PB10 IgG) was administered via intraperitoneal injection. This corresponded to an average of 39.9 μg/mL (± SD of 5.8) of Sal4 IgG present in the serum of mice at the time of challenge as determined by ELISA (samples from n = 3 mice). 24 h after antibody administration, mice were challenged orally with STm inoculum containing a 1:1 mixture of strains AR04 (mutant) and AR05 (wildtype). 24 h post-infection, mice were euthanized, and Peyer’s patches harvested and enumerated for CIs, as described in the Materials & Methods section. No statistical significance between the control and Sal4 IgG treatment groups was observed, as determined by unpaired Student’s t-test (P = 0.74). (TIF) [file pntd.0007803.s010.tif]
